# The POSH scaffold protein is essential for signal coordination leading to CD8 T cell differentiation and survival

**DOI:** 10.3389/fimmu.2025.1630599

**Published:** 2025-07-02

**Authors:** Caitlyn Guldenpfennig, Yue Guan, Bela Cseri, Elida Lopez, Emma Teixeiro, Mark Daniels

**Affiliations:** Department of Molecular Microbiology and Immunology NextGen Precision Health, University of Missouri, Columbia, SC, United States

**Keywords:** POSH, short-lived effector cell, memory precursors, signal transduction, NF-κB, Akt, JNK, CD8 T cell

## Abstract

**Introduction:**

Upon antigen recognition, naive CD8 T cells must induce c-JUN N-terminal kinase (JNK), NF-κB, and Akt signaling to drive differentiation and generate a heterogeneous effector response. While the roles of these three pathways individually in mediating essential cellular responses for CD8 T cell differentiation are well established, the mechanisms of signal integration and crosstalk between these pathways to produce a diverse and heterogeneous response to infection remain poorly understood. Here, we establish the critical role of the Plenty of SH3 Domains (POSH) scaffold protein in coordinating signals from all three pathways to support CD8 T cell differentiation and fate.

**Methods:**

Using novel conditional T cell POSH knockout reporter mouse models (as POSHfl/fl CD4-Cre eGFP, POSHfl/fl GzmB-Cre eGFP), we determined the phenotype of T cells in the thymus and periphery through flow cytometry. Polyclonal and OT1 TCR transgenic POSH cKO CD8 T cells were stimulated in vitro and analyzed by flow cytometry to assess cell fate. JNK, NF-κB, and Akt pathways were examined via flow cytometry and immunoblotting. Purified OT1 CD8 T cells from these mice were adoptively transferred and subsequently challenged with VSV-OVA infection; their phenotype, effector function, and signaling were then assessed ex vivo by flow cytometry.

**Results:**

We demonstrate that POSH is essential for proper induction of the JNK, NF-κB, and Akt pathways. Furthermore, the absence of these signals due to POSH deficiency results in reduced differentiation into short-lived effector cells (SLECs), delayed proliferation, and decreased survival of memory precursor cells (MPECs) during the contraction phase.

**Conclusions:**

Collectively, these data identify POSH as a key regulator of CD8 T cell fate and enhance our understanding of the complex mechanisms governing signal integration during CD8 T cell responses to infection.

## Introduction

CD8 T cells play a major role in the adaptive immune response to bacterial and viral infections. Signals initiated through antigen recognition by the TCR, co-stimulation, and cytokines cooperate to drive the activation and differentiation of naive CD8 T cells into a heterogeneous effector response composed of short-lived effector cells (SLECs), memory precursor (MPEC) CD8 T cells, and diverse subsets of long-lived memory cells (1). The multiple possible fates for a CD8 T cell are determined by a combination of factors, including the strength of TCR signaling, the presence or absence of co-stimulation, inflammation, cytokines, and the maturation state of the cell. These decisions are mediated by, or directed through, several signaling pathways, among which three of the most crucial are the JNK, NF-κB, and Akt pathways (2–4). Understanding how signals through these receptors and pathways are integrated to guide and determine these diverse cell fates is essential for improving the therapeutic application of CD8 T cells.

The JNK, NF-κB, and Akt signaling pathways each play integral and complex roles in the fate of CD8 T cells. In the thymus, JNK regulates negative selection (5), while in mature CD8 T cells, JNK signaling controls the upregulation of CD25, proliferation, differentiation, and effector function (4, 6, 7). NF-κB has a unique role throughout CD8 T cell ontogeny, including controlling survival and programming in the thymus (8, 9). In the periphery, NF-κB is important for supporting CD8 T cell differentiation, proliferation, and survival during activation, as well as playing a critical role in programming the differentiation and maintenance of various memory subsets (2, 10, 11). Sustained Akt activation promotes SLEC differentiation at the expense of MPECs and induces a transcriptional program that enhances effector functions (12–15). In contrast, Akt inhibition favors MPEC differentiation and results in an increased number of memory CD8 T cells (12, 14).

While each of these pathways has distinct roles in the CD8 T cell immune response, they also interact in complex ways. For example, both NF-κB and JNK can be activated by TNF-α, but NF-κB can also suppress JNK signaling downstream of TNF-α (16). Additionally, Akt activation can enhance NF-κB activity and increase NF-κB-dependent gene expression (17). Crosstalk and competition between Akt and JNK also regulate CD8 T cell survival (18). Therefore, these pathways not only act individually but also interact with and influence each other to ultimately regulate CD8 T cell fate and function. The mechanisms underlying the coordination of these complex interactions are not yet well understood.

Scaffold proteins serve as a means by which cells coordinate cellular signaling cascades. These proteins facilitate the formation of signaling nodes that mediate crosstalk between multiple pathways and act as modules for assembling components necessary for the activation of specific signaling cascades. They can also provide temporal and spatial regulation of these pathways, thereby directing and diversifying the potential outcomes of their activation (19–21).

One particular scaffold protein of interest is the Plenty of SH3 Domains (POSH) scaffold protein, as it has been shown to interact with components of the JNK, Akt, and NF-κB pathways. POSH is comprised of four SH3 domains, a Rac binding domain, and a RING finger domain with E3 ubiquitin ligase activity. POSH was first described as a Rac-binding protein that induced JNK and NF-κB signaling, leading to subsequent apoptosis in fibroblasts (22). Prior to our recent work on the function of POSH in T cells (7, 23), most of what was known about POSH’s role originated from studies in neurons, where it functions as a scaffold for the JNK signaling pathway (24). In mature neurons, assembly of the POSH/JNK complex leads to prolonged JNK signaling and apoptosis (25–27). However, its role in neuronal development differs; POSH is essential for the development of the cerebral neural network and neuronal outgrowth in developing fetuses (28). Given the unique role of JNK during thymocyte development and mature T cell activation, and considering POSH’s ability to interact with components of NF-κB and Akt signaling, we hypothesized that POSH may play a role in regulating CD8 T cell fate.

We and others have shown that multiple proteins associate with POSH (22, 24, 25) and that post-translational modifications of POSH are important in regulating the composition and function of the POSH signaling complex in T cells (7, 23, 29, 30). Using a competitive inhibitor of the third SH3 domain of POSH (SH3.3), we identified that the predominant scaffold complex in mature CD8 T cells contained Vav, Rac1, MLK3, MKK7, JIP-1, and JNK-1, with JIP-1 binding to SH3.3 (7). In this study, POSH inhibition led to the loss of JNK1 signaling, defects in IFN-γ production, delayed expression of CD25, and delayed proliferation. In CD4 T cells, we found that Rac2, Tak1, MKK7, JIP-1, and both JNK1/2 associated within the POSH signaling complex. Interestingly, in CD4 T cells, Tak1 was bound to SH3.3, and inhibition resulted in the loss of JNK1/2 signals, increased activation-induced cell death, and skewing toward a Th2 phenotype (23). Notably, we mapped these differences in composition to a phosphorylated tyrosine within the R1 binding loop of SH3.3 in CD8 T cells.

Others have shown that Akt can associate with POSH and negatively regulate its induction of JNK signaling, either through competitive binding or direct phosphorylation of binding domains within POSH (29, 30). Therefore, regulation of POSH-associated components adds an interesting layer of complexity to its function. While our previous work and others have largely focused on POSH SH3.3, a complete understanding of how the entire POSH scaffold assembly influences CD8 T cell differentiation and effector function remains to be elucidated.

To further understand the impact of POSH scaffold assembly on CD8 T cell fate, we developed conditional, T cell-specific POSH knockout models. We found that POSH is required for proper JNK and NF-κB–induced CD25 expression and Akt signaling, both essential for SLEC differentiation and survival *in vitro* and *in vivo*. Additionally, although POSH conditional knockout (POSH cKO) CD8 T cells predominantly differentiated into MPECs, these cells were unable to survive the contraction phase to form memory cells. This study demonstrates the vital role of POSH in coordinating JNK, NF-κB, and Akt signals to regulate CD8 T cell fate and advances our understanding of how cells utilize scaffolding proteins to modulate complex interactions among these pathways.

## Results

### Confirmation of POSH knockout

**Figure 1:**
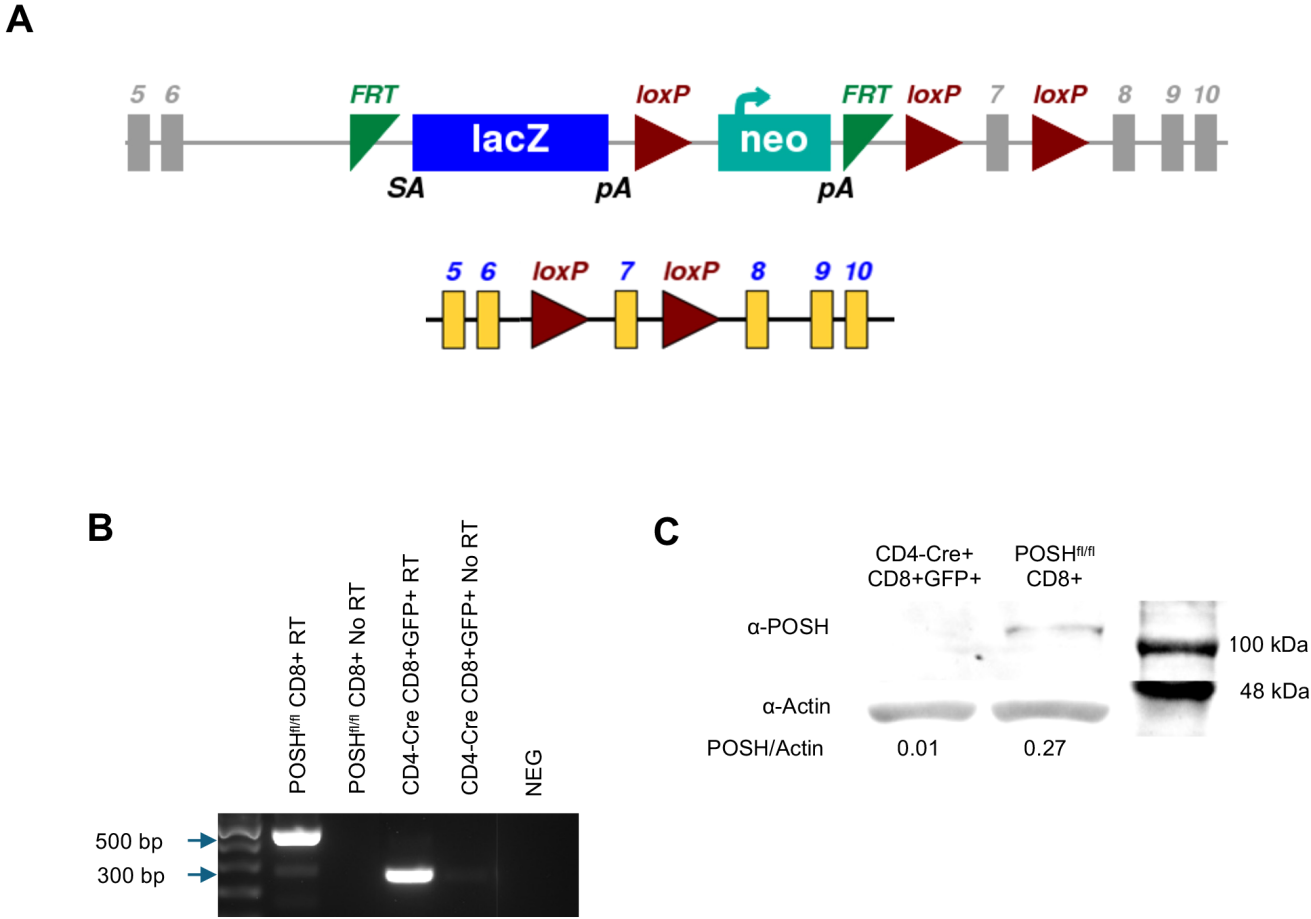
Confirmation of POSH deletion in POSH^fl/fl^ CD4-Cre mice. **(A)** Schematic depicting the original SH3rf1^tm1a(EUCOMM)Hmgu^ genetic construct (top) and the construct after deletion of the luciferase/neo cassette (bottom) for the POSH^fl/fl^ gene **(B, C)** GFP positive T cells were isolated, lysed, and mRNA and protein were isolated. **(B)** Deletion of exon 7 from the POSH mRNA was confirmed by RTPCR. (POSH mRNA with exon 7–500 bp, Δexon7 POSH mRNA – 300 bp) **(C)** Deletion of POSH at the protein level was confirmed by western blot.

POSH, expressed by the gene *sh3rf1*, is a scaffold molecule that contains four SH3 domains, a Rac binding domain, and multiple polyproline domains able to bind several signaling molecules important for T cell development and differentiation. We have recently demonstrated, using novel inhibitors, a role for the SH3 domain 3 (SH3.3) of POSH in regulating the activation of JNK and differentiation of T cells *in vitro* (7, 23). To develop a deeper understanding of the role of the full POSH molecule *in vivo*, we generated conditional knockout mice strains, POSH^fl/fl^ CD4-Cre and POSH^fl/fl^ GzmB-Cre, using the IMPC *Sh3rf1^tm1a(EUCOMM)Hmgu^* construct (**Figure 1A**, see also methods). These mice contained Stop^fl/fl^ Rosa26 eGFP cassettes to act as a reporter for POSH deficient T cells. The mice were maintained as homozygous for the POSH^fl/fl^ gene, homozygous for eGFP, and Cre positive (denoted as POSH^fl/fl^ CD4-Cre, POSH^fl/fl^ GzmB-Cre). POSH^fl/fl^ control strains that were Cre negative and Stop^fl/fl^ Rosa26 eGFP homozygous were also generated and are denoted as POSH^fl/fl^ or “Control”. POSH deficient GFP+ CD8 T cells are referred to as POSH cKO in the rest of the paper.

To confirm the deletion of POSH, CD8 T cells from POSH^fl/fl^ and POSH^fl/fl^ CD4-Cre mice were sorted by flow cytometry. RT-PCR confirmed that in POSH^fl/fl^ CD4-Cre mice, exon 7 is removed within CD8 T cells at the genetic level, resulting in a 300-base pair band (**Figure 1B**). At the protein level, POSH is undetectable in POSH^fl/fl^ CD4-Cre CD8 T cells as compared to POSH^fl/fl^ (**Figure 1C**). Thus, POSH is effectively knocked out in POSH^fl/fl^ CD4-Cre CD8 T cells.

### POSH has a minimal role in T cell development

POSH has been linked to JNK, Akt, and NF-κB signaling, and crosstalk between these pathways has been implicated in T cell fate in the thymus. JNK is important for negative selection in developing thymocytes and for effector function in mature cells (4, 5). Additionally, NF-κB differentially contributes to T cell development depending on the maturation stage, with CD8 T cell development being more dependent on NF-κB than CD4 (2, 8, 31). Akt and NF-κB interact to support survival and proliferation after TCRβ selection (3, 8).

**Figure 2:**
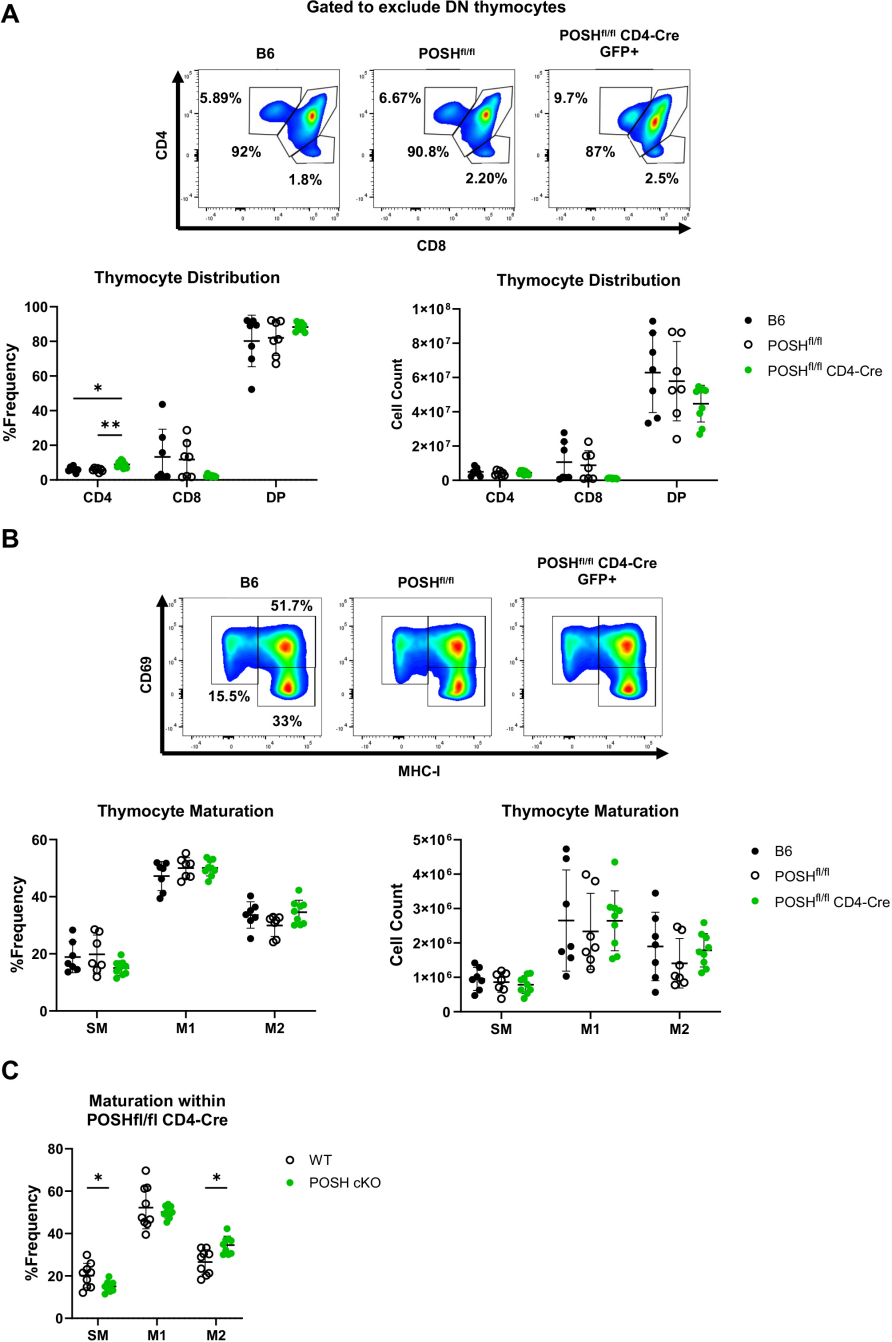
Polyclonal POSH^fl/fl^ CD4-Cre T cells have only minor defects in thymic development and maturation. **(A)** Representative plots (top), frequency (bottom left), and number (bottom right) of DP, CD4 and CD8 T cells within B6 (n=7), POSH^fl/fl^ (n=7) and POSH^fl/fl^ CD4-Cre (n=9) mice. **(B)** Representative plots (top), frequency (bottom left), and number (bottom right) of the stages of maturation in B6 (n=7), POSH^fl/fl^ (n=7) and POSH^fl/fl^ CD4-Cre (n=9) where SM is defined as CD69+MHC-I-, M1 as CD69+MCH-I+, and M2 as CD69-MCH-I+. **(C)** Frequency of POSH sufficient and POSH cKO T cells within each stage of maturation in POSH^fl/fl^ CD4-Cre mice (n=9). Data are shown as mean ± SD and are the combination of two independent experiments. Two-way ANOVA with the Geisser-Greenhouse correction and Tukey multiple comparisons **(A, B)** or Sidak’s multiple comparisons **(C)** test, with individual variances computed for each comparison was used to determine significance with *p<0.05, **p<0.001.

To define the role of POSH in T cell development, the phenotype of thymocytes from the thymi of polyclonal POSH^fl/fl^ CD4-Cre mice was examined by flow cytometry. POSH is deleted in these cells after TCRβ selection as they transition from CD4^-^, CD8^-^ double-negative (DN) thymocytes to cells expressing both CD4 and CD8, becoming CD4^+^, CD8^+^ double-positive (DP) thymocytes (32). Thus, our gating strategy excluded DN thymocytes and we compared the DP, CD8 single-positive (CD8SP), and CD4 single-positive (CD4SP) populations in the thymi of POSH^fl/fl^ CD4-Cre mice to those in C57BL/6 (B6) and POSH^fl/fl^ control mice (**Figure 2A**). While there are subtle differences in the number and frequency of DP, CD8SP, and CD4SP thymocytes, overall, these populations are similar between POSH^fl/fl^ CD4-Cre mice and controls. We additionally examined the maturation stages of single-positive thymocytes. Post-selection SP thymocytes must transition through the semi-mature (SM), mature 1 (M1), and mature 2 (M2) stages of maturation, which can be identified based on the expression of CD69 and MHC-I. This maturation process has been shown to be important for enabling cells to mount proliferative responses and activate effector functions upon antigen recognition before leaving the thymus (9). We found that medullary POSH cKO SP thymocytes appeared to mature normally compared to wild-type (WT) SPs from B6 and POSH^fl/fl^ controls (**Figure 2B**). However, when comparing the frequency of GFP^+^ POSH cKO thymocytes to GFP^-^ WT thymocytes from the same mouse, there were subtle but significant differences in the frequencies of SM and M2 cells (9), although there was no complete block in development (**Figure 2C**). Together, these results suggest that POSH, at least by itself, does not play a major role in T cell development or maturation.

### Phenotype of peripheral POSH cKO CD8 T cells in POSH^fl/fl^ CD4-Cre conditional mouse model

**Figure 3:**
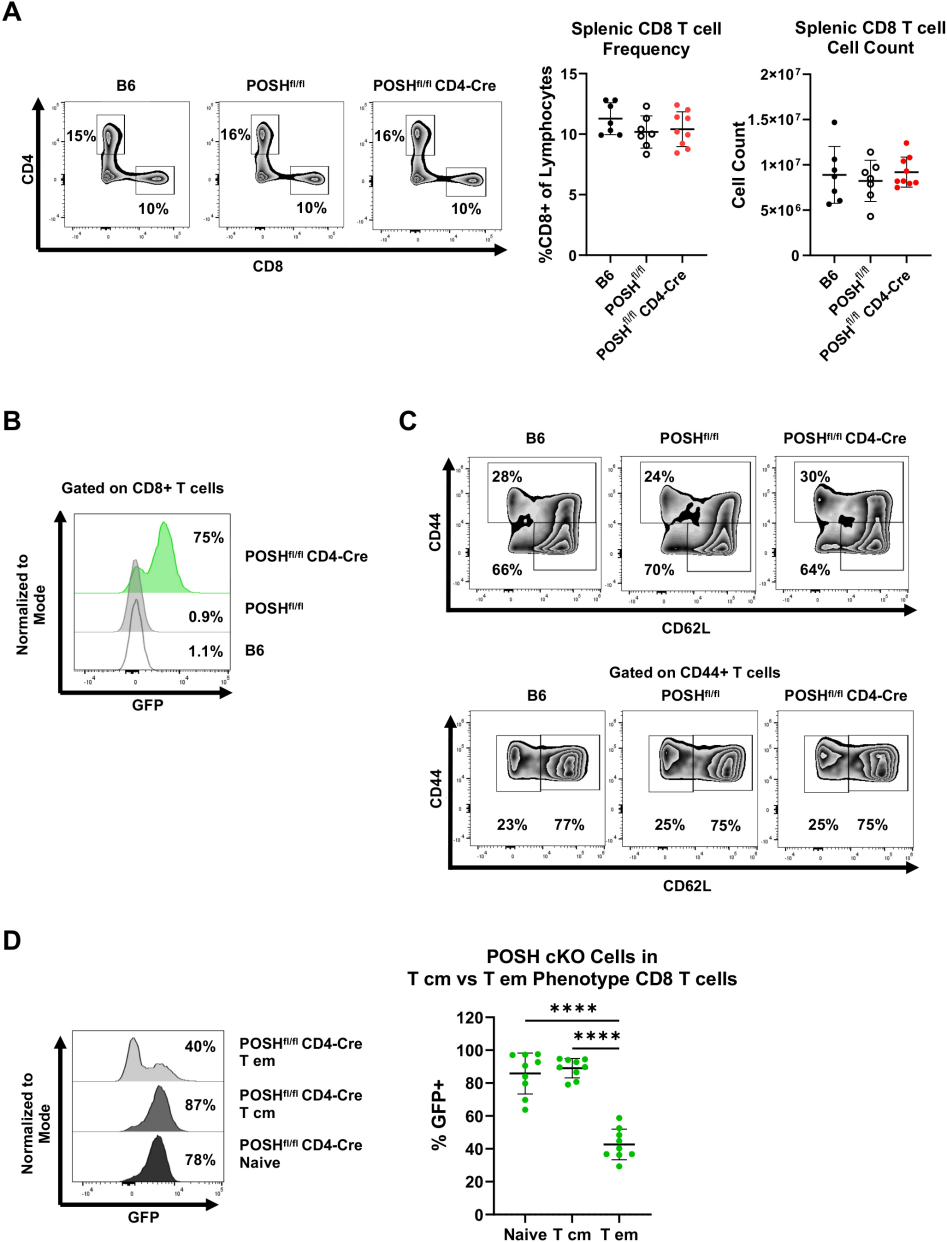
POSH cKO CD8 T effector/effector memory cells are lost in POSH^fl/fl^ CD4-Cre mice. **(A)** Representative plots (left) and quantification (right) of the frequency and number of CD8+ T cells within B6, POSH^fl/fl^, and POSH^fl/fl^ CD4-Cre mice. **(B)** Representative plot of the frequency of POSH cKO (GFP+) CD8+ T cells. **(C)** Representative plots depicting the frequency of naïve (CD44-CD62L+) and CD44 high CD8 T cells (top). Representative plots depicting T cm (CD44+CD62L+) and T effector/effector memory (CD44+CD62L-) CD8 T cells (bottom). **(D)** Representative plot (left) and quantification (right) of POSH cKO (GFP+) cells within the naïve, T cm, and T em CD8 T cell subsets in POSH^fl/fl^ CD4-Cre mice. Data are shown as mean ± SD and are the combination of 3 independent experiments with n=7 B6, n=7 POSH^fl/fl^, and n=9 POSH^fl/fl^ CD4-Cre. Ordinary one-way ANOVA with Tukey’s multiple comparison test, with a single pooled variance was used to determine significance with ****p<0.0001.

We next analyzed the phenotype of GFP^+^ POSH cKO CD8 T cells in the periphery of POSH^fl/fl^ CD4-Cre mice. There were no significant differences in the frequency or total number of CD8 T cells in the spleen (**Figure 3A**). However, within POSH^fl/fl^ CD4-Cre mice, only 75% of all splenic CD8 T cells are GFP^+^ POSH cKO T cells, with the remaining 25% being GFP^-^ WT CD8 T cells (**Figure 3B**). To determine which phenotype of POSH cKO cells was missing or lost, we examined the frequency of POSH cKO naïve and antigen-experienced CD8 T cells in these mice (**Figure 3C**). We found that approximately 85–90% of naïve CD8 T cells (CD44^-^ CD62L^+^) were POSH cKO (**Figure 3D**). Similarly, about 90% of T central memory (Tcm) (CD44^+^ CD62L^+^) CD8 T cells were POSH cKO (**Figure 3D**). However, within the T effector (Teff)/effector memory (Tem) CD8 population (CD44^+^ CD62L^-^), only 40% were POSH cKO (**Figure 3D**). A similar but less pronounced loss of POSH cKO cells was observed within the Teff/Tem CD4 T cell compartment, along with a significant reduction in all CD25^+^ POSH cKO CD4 T cells (**Supplementary Figure 1**). Collectively, these data suggest that the few GFP^-^ cells that did not delete POSH in these mice have a distinct advantage upon activation, providing strong support for the role of POSH in the differentiation, proliferation, and/or survival of activated CD8 T cells.

### POSH’s role in stimulated CD8 T cell differentiation

**Figure 4:**
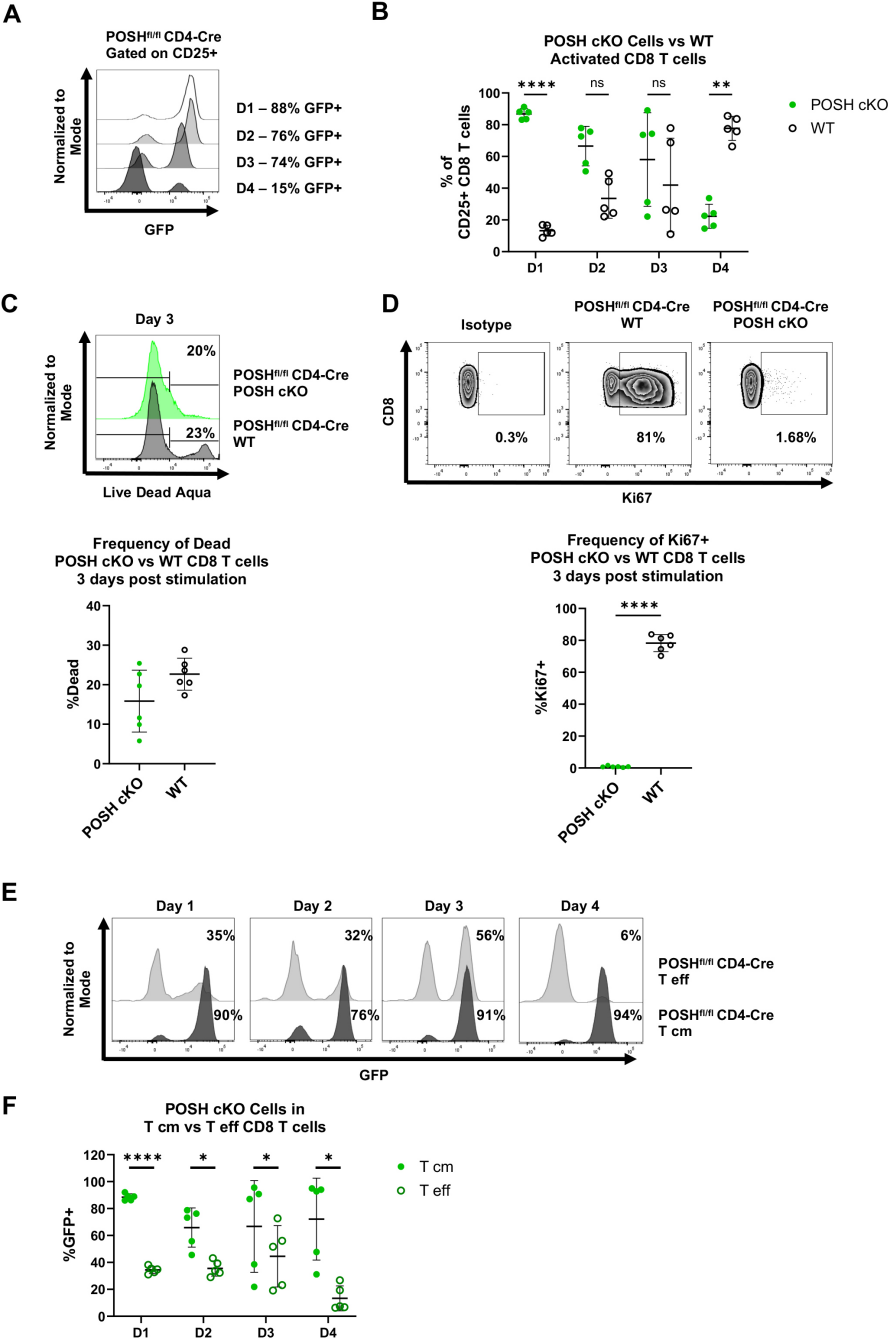
Activated T effector POSH cKO CD8 T cells are lost upon stimulation *in vitro*. Spleenocytes were isolated from POSH^fl/fl^ CD4-Cre mice and stimulated with αCD3/αCD28 for 4 days. Cells were given IL-2–24 hours post stimulation. **(A, B)** Representative plot **(A)** and quantification **(B)** of the frequency of POSH cKO (GFP+) vs WT (GFP-) cells gated on activated (CD25+) CD8 T cells on day 1–4 post stimulation. **(C)** Representative plot (top) and quantification (bottom) of the frequency of POSH cKO (GFP+) vs WT (GFP-) CD8 T cell death 3 days post stimulation. **(D)** Representative plots (top) and quantification (bottom) of the frequency of Ki67 expressing POSH cKO (GFP+) and WT (GFP-) CD8 T cells 3 days post stimulation. **(E, F)** Representative plots **(E)** and quantification **(F)** depicting the frequency of POSH cKO cells within the T eff and T cm subsets from POSH^fl/fl^ CD4-Cre mice on days 1–4 post stimulation. Data are shown as mean ± SD and are a combination of 2 independent experiments with n=5 POSH^fl/fl^ CD4-Cre. Multiple paired T test with Holm-Sidak’s multiple comparisons **(B, F)** or paired two-tailed T test **(C, D)** was used to determine significance with *p<0.05, **p<0.001, ****p<0.0001. ns, not significant.

The reduced number of POSH cKO CD8 T cells in the effector memory pool in POSH^fl/fl^ CD4-Cre mice led to the hypotheses that, upon stimulation, POSH cKO CD8 T cells are either unable to differentiate into effector or effector memory cells or are unable to survive following activation. To test this, we stimulated polyclonal CD8 T cells with α-CD3 and α-CD28 in the presence of IL-2 for 4 days and assessed their activation state (CD25) and differentiation state (CD44, CD62L) each day. There were no significant differences in the frequency or importantly, the number of CD8 T cells between the POSH^fl/fl^ CD4-Cre and control cultures after stimulation (data not shown). However, the percentage of CD25^+^ GFP^+^ POSH cKO CD8 T cells decreased each day, from 90% on day 1 to only 20% on day 4 (**Figures 4A, B**). In contrast, the GFP^-^ POSH WT CD8 T cells increased from 10% on day 1 to 80% on day 4 (**Figures 4A, B**).

Next, we examined whether the change in the frequency of POSH cKO and WT cells was due to defects in proliferation or apoptosis. Using a viability stain, we observed no significant difference in cell death between POSH cKO and WT cells (**Figure 4C**). However, significantly fewer POSH cKO cells expressed Ki-67, a marker of proliferation, compared to WT CD8 T cells (**Figure 4D**), indicating a proliferation defect in POSH cKO cells. These findings are consistent with previous results obtained using our POSH inhibitor (7).

Given the phenotype of POSH cKO cells in the periphery, we also assessed the frequency of CD8 Tcm precursors (CD44^+^ CD62L^+^) and CD8 Teff (CD44^+^ CD62L^-^) cells *in vitro*. Notably, the frequency of POSH cKO Teff cells was significantly reduced at day 4, while POSH WT Teff cells continued to increase and comprised the majority of the CD8 T cell population (**Figure 4E**). Although the frequency of POSH cKO Tcm cells decreased slightly over time, the remaining cells at day 4 more often exhibited the Tcm phenotype (**Figure 4F**). Due to the decrease in POSH cKO Teff cells, we next evaluated their effector functions. There was a slight but statistically significant decrease in killing capacity, likely not to be physiologically or biologically relevant (**Supplementary Figure 2**). Additionally, fewer POSH cKO cells expressed Granzyme B, but there was no difference in the frequency of IFN-γ-expressing POSH cKO cells (**Supplementary Figure 3**). Thus, while POSH cKO Teff cells were capable of acquiring effector functions and being recruited into the effector pool early, they exhibited reduced proliferation and Teff differentiation *in vitro* compared to WT CD8 T cells.

### POSH cKO CD8 T cells and JNK, NF-κB, and Akt signaling

**Figure 5:**
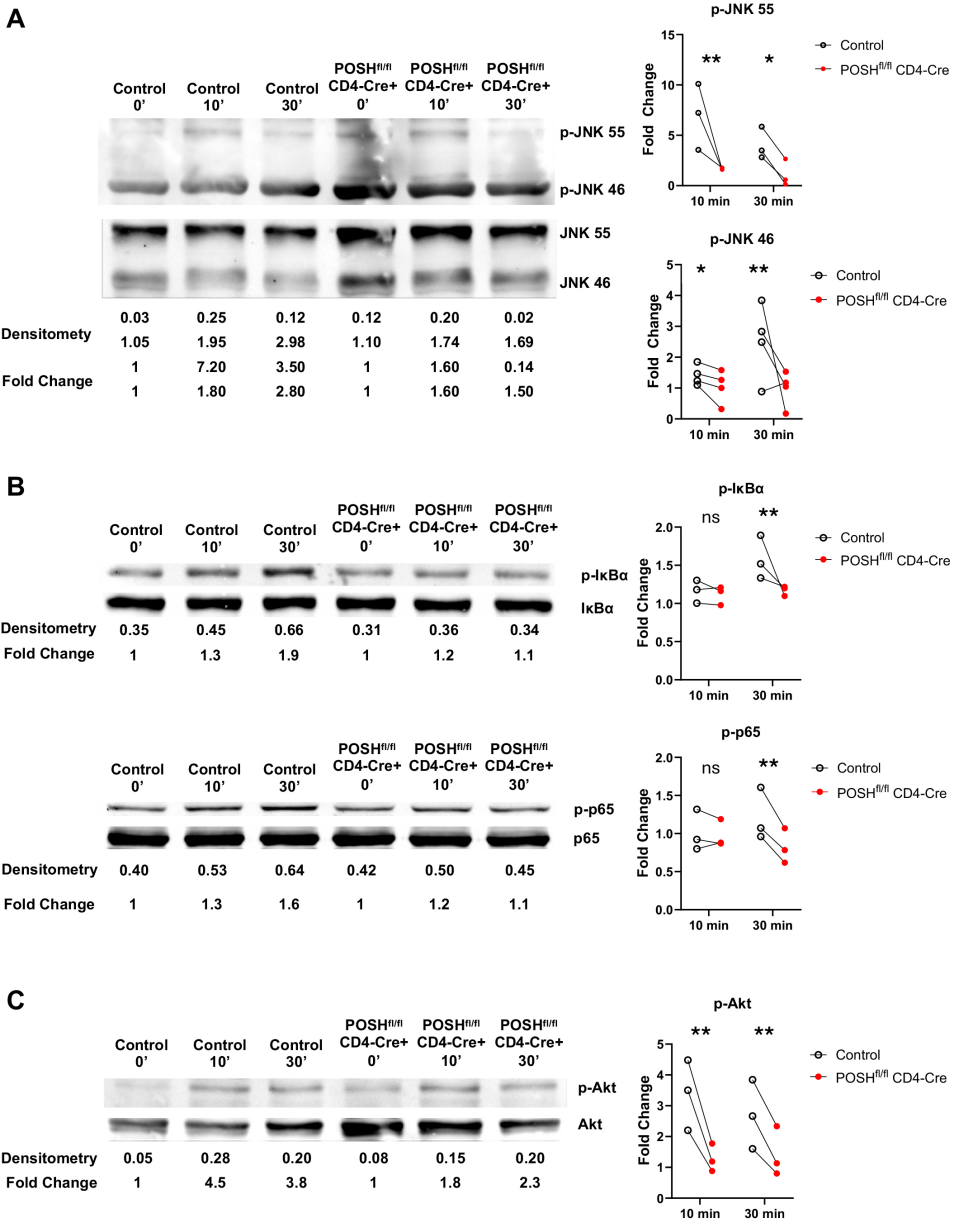
POSH^fl/fl^ CD4-Cre CD8 T cells have decreased JNK, NF-κB and Akt signaling. Control and POSH^fl/fl^ CD4-Cre CD8+ T cells were isolated via magnetic bead negative selection and stimulated with PMA/Ionomycin **(A, C)** or αCD3/αCD28 **(B)** for 0, 10, and 30 minutes. **(A)** Representative blot depicting levels of p-JNK 55 and p-JNK 46 (left) and quantification of fold change (right). **(B)** Representative blot depicting levels of p-IκBα (top left) or p-p65 (bottom left) and quantification of fold change (right) **(C)** Representative blot depicting levels of p-Akt (left) and quantification of fold change (right). Densitometry was calculated by normalizing the ROI of the phosphorylated protein to that of the total protein (ex. [ROI of p-JNK 55]/[ROI of JNK 55]). Fold change was calculated by normalizing the densitometry values to the 0 minute densitometry value (ex. [densitometry of Control 10 min]/[densitometry of Control 0 min]). Data are shown as each dot representing one western blot and lines connecting control and POSH^fl/fl^ CD4-Cre from the same western blot. A combination of 3–4 independent experiments are shown for each protein. Two-way ANOVA/Mixed Effect Model was used to determine significance with *p<0.05, **p<0.001.

Given the observed defects during *in vitro* differentiation (**Figure 4**) and the lack of activated POSH cKO CD8 T cells (Teff/Tem) in the periphery of POSH^fl/fl^ CD4-Cre mice (**Figure 3**), we hypothesized that these results were linked to defects in JNK, NF-κB, and/or Akt signaling. To examine these potential defects, we purified and stimulated GFP^+^ POSH cKO CD8 T cells from POSH^fl/fl^ CD4-Cre mice and analyzed signaling components by Western blot. We found that phosphorylation of both JNK1 and JNK2 was decreased in CD8 T cells compared to controls (**Figure 5A**). Additionally, defects in the NF-κB pathway were evident, as phosphorylation of IκBα and p65 were also markedly reduced (**Figure 5B**). Finally, phosphorylation of Akt at Serine 473 was significantly diminished upon stimulation (**Figure 5C**).

Thus, all three major pathways associated with POSH function are reduced in POSH cKO CD8 T cells. These findings differ subtly from those observed with POSH SH3.3 inhibition, where no defect in NF-κB or Akt signaling was detected. This highlights the importance of the remaining protein-binding domains within the scaffold and provides insight into their essential roles in POSH function in CD8 T cells.

### POSH cKO CD8 T cells response to viral infection *in vivo*

So far, our data suggest that POSH plays an important role in the differentiation and proliferation of CD8 T effector subsets *in vitro*. To further assess the role of POSH in CD8 T cell differentiation, we next tested the ability of POSH cKO CD8 T cells to differentiate in response to viral infection *in vivo*. To do this, we adoptively transferred OT1 POSH^fl/fl^ (OT1 control) or OT1 POSH^fl/fl^ CD4-Cre T cells into congenically marked host mice, infected them with VSV-OVA, and tracked antigen-specific donor CD8 T cells in the blood over the course of the immune response.

**Figure 6:**
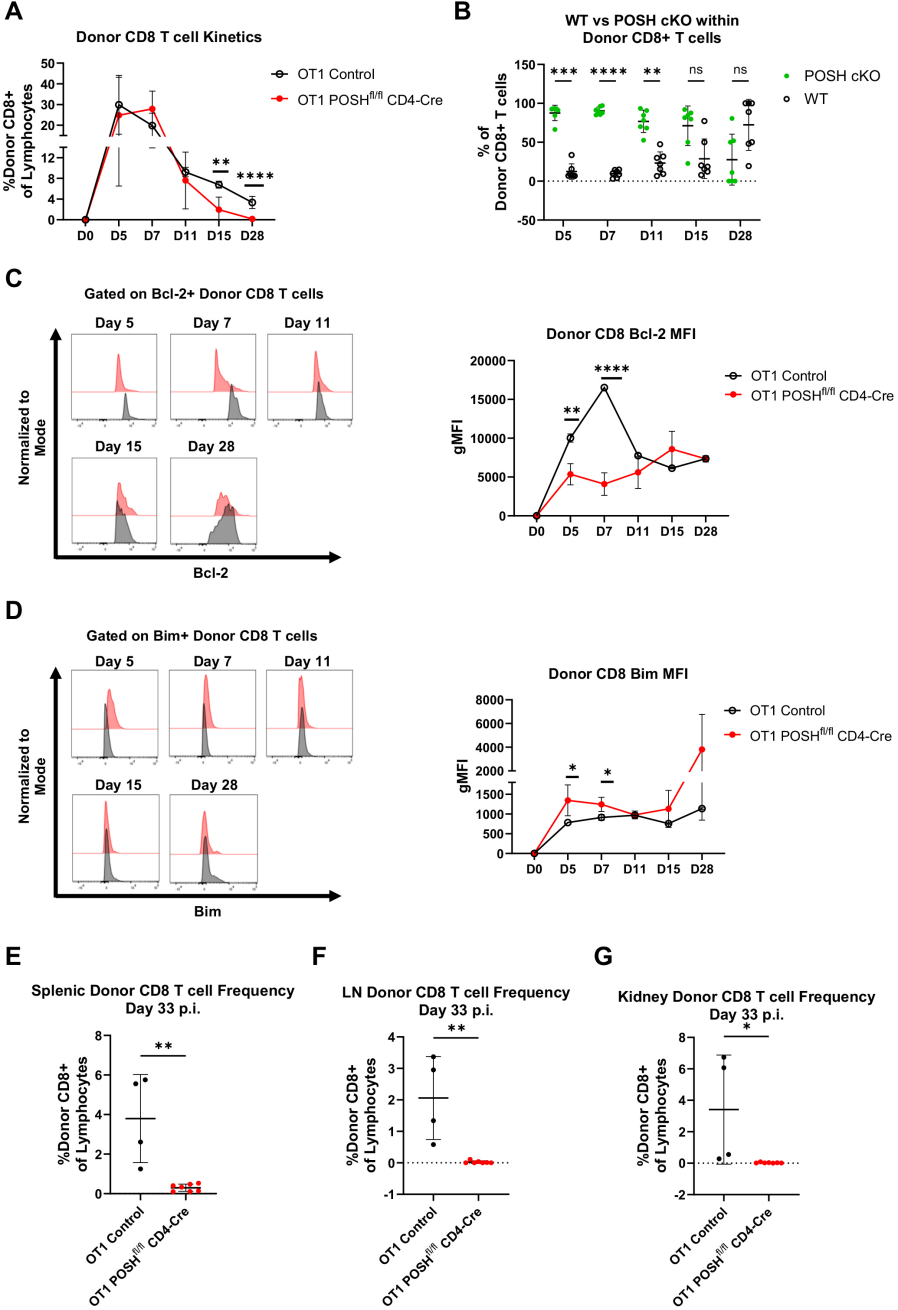
POSH cKO donor cells are not equipped to survive into the memory phase post VSV-OVA infection. 100,000 OT1 Ly5.2 Control or OT1 Ly5.2 POSH^fl/fl^ CD4-Cre CD8 T cells were adoptively transferred into Ly5.1 B6 hosts. 24 hours later mice were injected i.v. with VSV-OVA. Tail bleeds were performed 5, 7, 11, 15, and 28-days post infection. 33 days post infection, mice were humanely euthanized and spleen, lymph node (LN) and kidney were harvested. **(A)** Frequency of Ly5.2 CD8+ donor cells in the blood post VSV-OVA infection. n=4 OT1 Control and n=7 OT1 POSH^fl/fl^ CD4-Cre. **(B)** Frequency of POSH cKO (GFP+) vs WT (GFP-) Ly5.2 CD8+ donor cells of mice adoptively transferred with OT1 POSH^fl/fl^ CD4-Cre cells (n=7). **(C, D)** Representative plots (left) and quantification (right) of the gMFI of Bcl-2 **(C)** and Bim **(D)** within Ly5.2 CD8+ donor cells post VSV-OVA infection. n=2 OT1 Control and n=7 POSH^fl/fl^ CD4-Cre. **(E-G)** Frequency of Ly5.2 CD8+ donor cells in the spleen **(E)**, LN **(F)**, and kidney **(G)** 33 days post VSV-OVA infection. n=4 OT1 Control and n=7 OT1 POSH^fl/fl^ CD4-Cre. Data are shown as mean ± SD and are the combination of 5 independent experiments. Significance was determined using **(A)** Multiple unpaired T test with Holm-Sidak multiple comparisons test **(B)** Multiple paired T test with Holm-Sidak’s multiple comparisons **(C, D)** Mixed-effects model with the Geisser-Greenhouse correction and Sidak’s multiple comparisons test with individual variances computed for each comparison or **(E-G)** Unpaired two-tailed T test with *p<0.05, **p<0.001, ***p<0.0002, ****p<0.0001. ns, not significant.

Interestingly, POSH^fl/fl^ CD4-Cre T cells showed a slight delay in their ability to expand in response to VSV-OVA infection compared to controls, as the peak response shifted from day 5 to day 7 (**Figure 6A**). However, the donor OT1 POSH^fl/fl^ CD4-Cre CD8 T cells were significantly reduced by days 15 and 28 post-infection (**Figure 6A**). Correspondingly, we observed a decrease in the frequency of POSH cKO cells and an increase in WT POSH-sufficient donor cells on days 15 and 28 post-infection in mice transferred with OT1 POSH^fl/fl^ CD4-Cre CD8 T cells (**Figure 6B**). This suggests that POSH cKO CD8 T cells are less capable of survival.

The balance between Bcl-2 (pro-survival) and Bim (pro-apoptotic) molecules is critical for the survival of both effector and effector memory precursors (33–36). Therefore, we monitored their levels throughout the *in vivo* response and found that OT1 POSH cKO cells expressed significantly lower levels of Bcl-2 and higher levels of Bim on days 5 and 7 compared to OT1 controls (**Figures 6C, D**). This Bim/Bcl-2 phenotype is associated with cell death and likely results from defects in NF-κB (10) and Akt signaling (**Figure 5**). Supporting this, at the endpoint of the experiment, we found very few OT1 POSH cKO donor T cells in the spleen (**Figure 6E**), and nearly none in the lymph nodes (**Figure 6F**) or kidneys (**Figure 6G**). Taken together, in addition to the apparent defects in survival, these findings also indicate that the loss of POSH cKO CD8 T effector/effector memory cells (**Figure 3**) was likely not due to a lack of CD4 T cell help.

To corroborate these findings and to determine whether POSH availability during early activation could rescue differentiation, we performed the same adoptive transfer experiment using OT1 POSH^fl/fl^ GzmB-Cre mice. These mice were generated in the same manner as described above, and POSH knockout was confirmed by day 3 post-stimulation (**Supplementary Figure 4**, data not shown). As before, OT1 Control or OT1 POSH^fl/fl^ GzmB-Cre CD8 T cells were transferred into congenically marked hosts followed by VSV-OVA infection. Similar to what we observed with OT1 POSH^fl/fl^ CD4-Cre CD8 T cells, the peak response of OT1 POSH^fl/fl^ GzmB-Cre donor CD8 T cells shifted from day 5 to day 7 (**Supplementary Figure 4A**). However, by day 28, the OT1 POSH cKO donor CD8 T cells were again lost from circulation, similar to the POSH cKO cells in OT1 POSH^fl/fl^ CD4-Cre mice (**Figure 6**, **Supplementary Figure 4A**). Additionally, POSH cKO cells failed to outcompete WT POSH cells in mice adoptively transferred with OT1 POSH^fl/fl^ GzmB-Cre cells (**Supplementary Figure 4B**). Similarly, there was a significant decrease in Bcl-2 expression and a notable increase in Bim within OT1 POSH cKO donor CD8 T cells (**Supplementary Figures 4C, D**). The OT1 POSH cKO CD8 T cells were also not detected in the spleen, lymph nodes, or kidneys of the recipient mice (**Supplementary Figures 4E–G**). Thus, POSH cKO CD8 T cells, regardless of when POSH expression is lost, are unable to survive into the memory phase following VSV infection.

**Figure 7:**
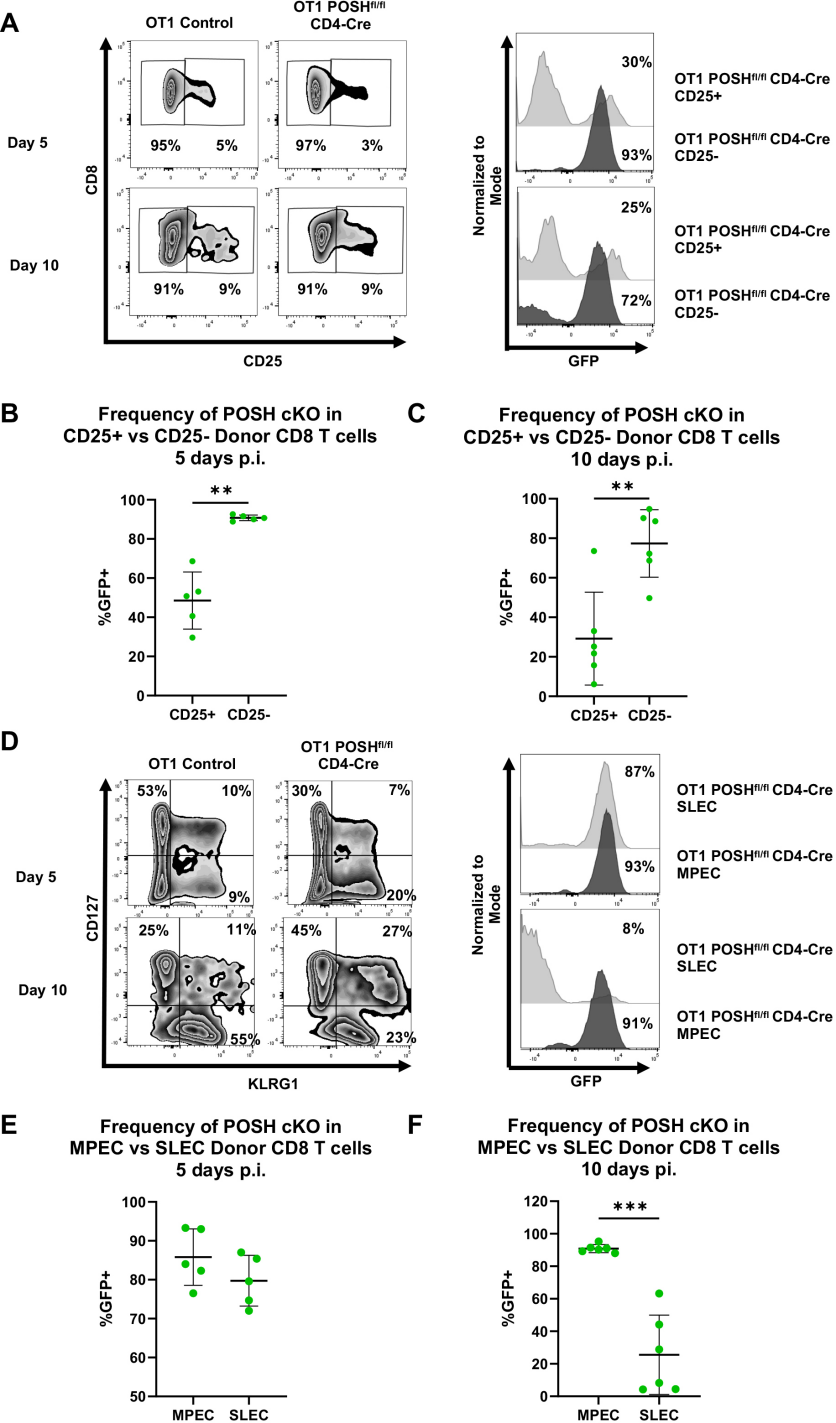
CD25+ POSH cKO cells are significantly reduced *in vivo*. 100,000 OT1 Ly5.2 Control or OT1 Ly5.2 POSH^fl/fl^ CD4-Cre CD8 T cells were adoptively transferred into Ly5.1 B6 hosts. 24 hours later mice were injected i.v. with VSV-OVA. On day 5 or day 10 post infection, mice were humanely euthanized, and spleens were harvested for analysis. **(A)** Representative plots depicting frequency of CD25- and CD25+ CD8+ donor cells on day 5 and day 10 post infection (left). Representative plots depicting the frequency of POSH cKO (GFP+) cells within the CD25- and CD25+ CD8+ donor subsets of mice adoptively transferred with OT1 POSH^fl/fl^ CD4-Cre cells on days 5 and 10 post infection (right). B-C) Quantification of the frequency of POSH cKO (GFP+) cells within the CD25- and CD25+ CD8+ donor subsets of mice adoptively transferred with OT1 POSH^fl/fl^ CD4-Cre cells 5 days **(B)** or 10 days **(C)** post infection. **(D)** Representative plots depicting frequency of MPEC (CD127+KLRG1-) and SLEC (CD127-KLRG1+) CD8+ donor cells on day 5 and day 10 post infection (left). Representative plots depicting the frequency of POSH cKO (GFP+) cells within the SLEC and MPEC CD8+ donor subsets of mice adoptively transferred with OT1 POSH^fl/fl^ CD4-Cre cells on days 5 and 10 post infection (right). **(E, F)** Quantification of the frequency of POSH cKO (GFP+) cells within the MPEC and SLEC CD8+ donor subsets of mice adoptively transferred with OT1 POSH^fl/fl^ CD4-Cre cells 5 days **(E)** or 10 days **(F)** post infection. Data are shown as mean ± SD and the combination of 2 independent experiments with n=5 OT1 POSH^fl/fl^ CD4-Cre mice at each time point. Paired two-tailed T test was used to determine significance with **p<0.001, ***p<0.0002.

Given the loss of T effector cells observed *in vitro* and the reduction of memory cells *in vivo* (**Figures 4**, **6**), we assessed the activation and differentiation status of antigen-specific donor CD8 T cells in response to VSV-OVA infection. We found that, at both day 5 and day 10 post-infection, there was a significant reduction in the frequency of CD44^+^ CD25^+^ POSH cKO CD8 T cells compared to CD44^+^ CD25^-^ POSH cKO cells (**Figures 7A–C**). This finding aligns with the observed defects in JNK and NF-κB signaling (**Figure 5**).

Importantly, CD25 and IL-2 are essential for SLEC differentiation via the Akt/mTOR pathway, and loss of these signals has been shown to increase MPEC differentiation (12, 14, 15, 37–39). Based on the loss of CD25 expression and the significant reduction in Akt phosphorylation in POSH cKO cells, we hypothesized that POSH cKO CD8 T cells would exhibit decreased SLEC and increased MPEC differentiation. This is supported by our data showing that, by day 10 post-infection, POSH cKO CD8 T cells within the SLEC population were significantly reduced, with these cells primarily differentiating into MPECs (**Figures 7D–F**). Collectively, the lack of CD25 expression and defects in Akt signaling (**Figure 5**) suggest that impaired CD25 signaling through Akt contributed to defective SLEC differentiation and survival. These findings highlight a role for POSH in coordinating JNK, NF-κB, and/or Akt signals that support proper SLEC differentiation.

Finally, due to the impaired Akt signaling in POSH cKO cells and the decreased SLEC differentiation, we examined downstream activity of the Akt/mTOR pathway, which is known to be essential for T-bet expression and SLEC differentiation (12, 14, 15). Given the reduced SLEC and increased MPEC differentiation observed, we hypothesized that POSH cKO CD8 T cells would exhibit decreased T-bet expression and p-S6, a downstream marker of mTOR activity, along with increased Eomes expression.

**Figure 8:**
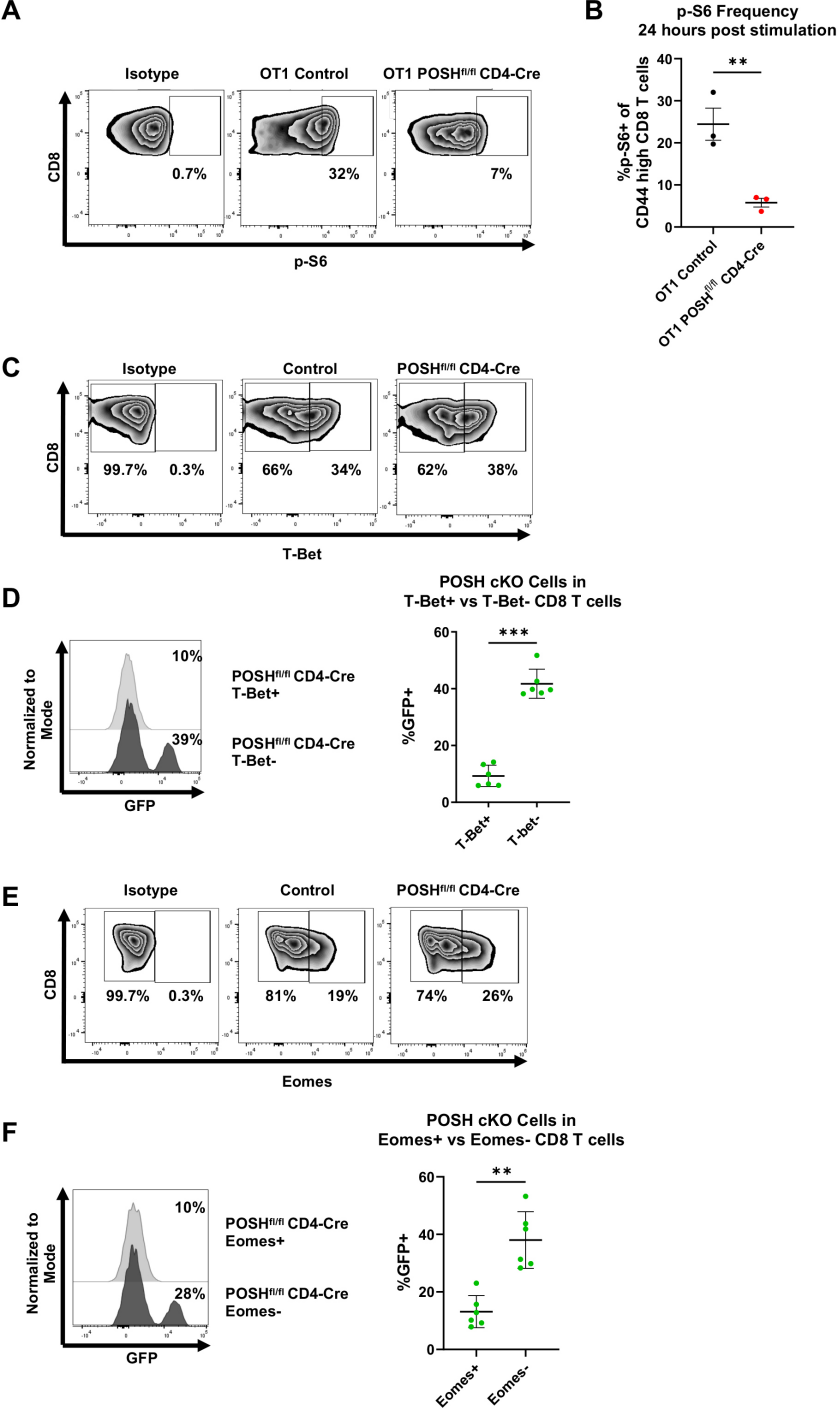
POSH cKO CD8 T cells have decreased p-S6, T-Bet, and Eomes expression. **(A, B)** OT1 Control and OT1 POSH^fl/fl^ CD4-Cre CD8 T cells were stimulated with OVA peptide and 24 hours later p-S6 expression was measured by flow cytometry. Representative plots **(A)** and quantification **(B)** of p-S6 expression are shown with n=3 for each group. **(C-F)** Control and POSH^fl/fl^ CD4-Cre CD8 T cells were stimulated with αCD3/αCD28 for 4 days. **(C)** Representative plots depicting T-Bet expression on day 4 post stimulation. **(D)** Representative plot (left) and quantification (right) of the frequency of POSH cKO cells within the T-Bet+ and T-Bet- subsets in POSH^fl/fl^ CD4-Cre cultures (n=6). **(E)** Representative plots depicting Eomes expression on day 4 post stimulation. **(F)** Representative plot (left) and quantification (right) of the frequency of POSH cKO cells within the Eomes+ and Eomes- subsets in POSH^fl/fl^ CD4-Cre cultures (n=6). Data are shown as mean ± SD and are the combination of 2 independent experiments. Unpaired, two-tailed T test **(B)** or paired, two-tailed T test **(D, F)** were used to determine significance with **p<0.001, ***p<0.0002.

We found that, 24 hours post-stimulation *in vitro*, POSH cKO CD8 T cells had significantly lower levels of p-S6 compared to control CD8 T cells (**Figures 8A, B**). There was also a significant decrease in T-bet^+^ POSH cKO CD8 T cells (**Figures 8C, D**), confirming their impaired differentiation into T effector cells. Interestingly, Eomes^+^ POSH cKO CD8 T cells were also reduced in POSH^fl/fl^ CD4-Cre cultures (**Figures 8E, F**). While unexpected, the reduced Eomes expression—likely mediated by the observed NF-κB defects (**Figure 5**)—may explain the poor survival of the early-formed MPEC population during the immune response (2, 10). Together, these results suggest that POSH is critical for coordinating JNK, NF-κB and Akt/mTOR signaling pathways that promote SLEC and MPEC differentiation, proliferation, and survival.

## Discussion

In this study, we utilized our novel POSH cKO mouse models to identify the necessity of POSH expression in CD8 T cells for regulating signals through the JNK, NF-κB, and Akt pathways that promote CD8 T cell differentiation, proliferation, and survival. We showed that, while POSH cKO cells are able to upregulate and express CD25 early on, due to defects in JNK and NF-κB signaling, they are unable to maintain its expression. This diminishes their ability to respond to IL-2 signaling and inhibits their proliferation and survival. We also demonstrate that POSH cKO CD8 T cells exhibit reduced induction of the Akt/mTOR pathway, as evidenced by decreased expression of T-Bet and reduced phosphorylation of the S6 protein. Consequently, they fail to activate the signals required for full differentiation into short-lived effector cells. Interestingly, although POSH cKO cells differentiate into MPECs early, they fail to express Eomes, likely due to defects in NF-κB signaling. Additionally, the Bim to Bcl-2 ratio in activated POSH cKO CD8 T cells—potentially linked to JNK/Akt and/or NF-κB pathways—favors apoptosis early in the response. Ultimately, POSH cKO CD8 T cells do not survive into the memory phase post-infection. Taken together, these data indicate that POSH is essential for CD8 T cell differentiation, proliferation, and survival, and plays a significant role in the ability of cells to survive contraction and enter the memory phase.

The loss of POSH has a significant effect on the PI3K/Akt/mTOR pathway. This signaling pathway is essential for the differentiation of SLECs (18). T cells subjected to rapamycin-based inhibition of mTOR exhibit increased memory generation, characterized by higher expression of CD127 and CD62L, and decreased KLRG1 expression (12). Others have shown that inhibition of mTOR activity leads to decreased T-Bet expression and promotes the generation of memory precursors (15). Furthermore, sustained activation of Akt results in increased mTOR activity, which enhances SLEC differentiation and inhibits MPEC formation (14). Our data corroborate these findings and demonstrate POSH’s role in coordinating these signaling events, as POSH cKO cells show decreased Akt/mTOR signaling, leading to reduced SLEC and increased MPEC differentiation both *in vitro* and *in vivo*. This also confirms POSH’s connection to Akt in CD8 T cells. In tumors, Akt1 and Akt2 have been shown to associate with POSH, negatively regulating the assembly of the JNK signaling complex by either competitively binding directly to POSH or by phosphorylating POSH to inhibit the binding of signaling components crucial for the JNK pathway (29, 30). Our work extends the connection between POSH and Akt, suggesting that POSH can also regulate Akt/mTOR signaling, although the precise mechanism remains to be determined.

Defects in JNK (7, 23) and NF-κB (8) can explain the abnormalities observed here in CD25 expression. Many studies have highlighted the importance of common gamma chain (γc)-containing receptors, such as the CD25/IL-2 receptor, in CD8 T cell differentiation into SLECs. Cells that express low levels of CD25 primarily differentiate into memory precursors, whereas cells with high IL-2R expression give rise to KLRG1^hi^ and T-bet^+^ SLECs (40, 41). However, it remains unclear whether the loss of SLEC differentiation seen here is solely due to an inability of POSH cKO cells to maintain CD25 expression or if POSH additionally coordinates essential Akt signals downstream of the IL-2 receptor (42).

Importantly, the decrease in MPECs during the contraction phase of the immune response cannot be explained solely by a lack of CD25 expression, as previous studies in CD25 knockout mice or IL-2/IL-15 receptor knockout mice show that CD8 T cells unable to signal through the IL-2 receptor can still generate functional memory populations (38–40). Interestingly, defects in NF-κB and Eomes have also been linked to failures in MPEC survival and memory differentiation (2, 11, 43). POSH may also coordinate signals downstream of other γc receptors to regulate CD8 T cell survival during contraction, as the phenotype of activated POSH cKO CD8 T cells resembles that of a complete γc receptor knockout model (37). Since CD25, IL-7, and IL-15 can all signal through NF-κB and Akt, it is intriguing to consider that POSH may coordinate downstream NF-κB and Akt signaling from these receptors to promote MPEC differentiation or survival. However, further studies are required to confirm this.

Historically, POSH function has been primarily associated with JNK signaling, with phenotypes upon POSH loss resembling those observed with JNK signaling deficiency itself (7, 23, 25, 44, 45). While earlier studies focused on POSH SH3.3, a key point from this study is that, although POSH may be involved in inducing JNK signaling, deletion of POSH does not always produce phenotypes solely consistent with JNK inhibition. Moreover, POSH deletion affects other pathways as well. Similar to our previous studies using a competitive inhibitor of POSH SH3.3, POSH cKO CD8 T cells displayed proliferation defects *in vitro*. However, IFN-γ expression remained normal in POSH cKO CD8 T cells. Our earlier work with the inhibitor led to a near-complete block in JNK1 signaling (7), whereas here, loss of POSH affected both JNK1 and JNK2, albeit to a lesser extent. Crosstalk or imbalance between these two JNKs could compensate for certain defects or, conversely, induce apoptosis (6, 46).

Additional factors may also account for discrepancies between these studies. Our previous inhibitor-based approach targeted the domain of POSH that blocks JIP-1 and JNK-1 binding but left other domains and proteins unaffected, which may explain differences in outcomes. For instance, in CD4 T cells, the Tat-POSH SH3.3 domain inhibitor prevented Tak1 from binding to POSH, while JIP and JNK associated with separate domains (23). Consequently, major signaling components could still bind POSH when treated with the inhibitor, but the complete knockout of POSH prevents their assembly altogether. Furthermore, inhibitor-based disruption of POSH complexes in CD4 and CD8 T cells did not significantly impact Akt or NF-κB signaling (7, 23), suggesting that the domains regulating crosstalk between JNK, Akt, and NF-κB do not involve SH3 domain 3. In fact, NF-κB signaling via POSH has been linked to SH3 domain 4 in fibroblasts (22, 44, 47, 48). Therefore, dissecting the roles of POSH’s various domains has helped reveal the diverse, context-dependent functions of POSH within cells.

Given the role of JNK/Akt/NF-κB in thymocyte development, the mild effect observed in POSH-deficient thymocytes was an interesting surprise. As stated earlier, Akt and NF-κB have been linked to survival during the transition from double-negative (DN) to double-positive (DP) thymocytes. Since Cre activation initiates POSH deletion at this stage, the lack of effect may be due to residual POSH present in the cells. The absence of an effect during the DP stage might also be due to differences in the POSH complex itself. In pre-selection thymocytes, POSH is phosphorylated in the Rac-binding domain, which blocks Rac from associating with the complex (7, 30), theoretically preventing its ability to influence JNK and possibly NF-κB signaling. This maturation stage-specific change in POSH function has also been observed in neurons (28).

Additionally, Tak1 and NF-κB have been linked to later stages of thymocyte maturation and development (9). Although we observe subtle differences in POSH cKO medullary thymocyte frequencies and numbers, there is no physiologically significant block in development or maturation. Moreover, there is no apparent change in the functional programming of these cells, as demonstrated by the results obtained from POSH cKO T cells in the POSH^fl/fl^ GzmB-Cre mice, which delete POSH only after activation. It remains an open question whether POSH is involved in regulating the JNK/Akt/NF-κB axis in the thymus or if redundant mechanisms coordinate these signaling cascades; this will be the focus of future studies.

In conclusion, this study identified the necessity of POSH expression within CD8 T cells for coordinating signals through JNK, NF-κB, and Akt/mTOR pathways during SLEC differentiation and memory CD8 T cell formation. This has potential broader implications, particularly in contexts where targeting short-term effector cells could be therapeutically beneficial, such as in autoimmune diseases. Furthermore, the complex regulation of multiple essential signaling pathways by POSH suggests it could be an ideal candidate for therapeutic targeting to modify crosstalk between the Akt, NF-κB, and JNK pathways. Advancing our understanding of POSH and its role in signaling within CD8 T cells will contribute to a more comprehensive understanding of T cell differentiation and may provide new strategies for manipulating this process to improve treatments for autoimmune diseases and cancer.

## Methods

### Mice

POSH^fl/fl^ mice were generated by the University of Missouri Transgenic Mouse facility from Sh3rf1^tm1a(EUCOMM)Hmgu^ KO first allele (reporter-tagged insertion with conditional potential) from the International Mouse Phenotype constortium (IMPC). Transgene positive mice were crossed with FLPo mice (Jackson Labs) to delete FRT flanked luciferase/neo cassette and backcrossed >9 generations to create POSH^fl/fl^ mice (**Figure 1A**).

POSH^fl/fl^ mice were then crossed with Rosa26-STOP-eGFP, CD4-Cre mice, GzmB-Cre and OT-I mice (all strains: Jackson Labs) and are kept homozygous for POSH^fl/fl^ and Rosa26-STOP-eGFP alleles, and hemizygous for Gzmb-Cre, CD4-Cre and OT-I alleles. Mice were maintained in our animal facilities at the University of Missouri. Animal procedures were in accordance with Intuitional Animal Care and Use Committee regulations.

### POSH knockout confirmation

Spleens from OT1 POSH^fl/fl^, OT1 POSH^fl/fl^ CD4-Cre, and OT1 POSH^fl/fl^ GzmB-Cre were harvested and processed into single cell suspensions. For POSH^fl/fl^ and POSH^fl/fl^ CD4-Cre, cells were stained with α-CD8 and CD8+GFP- or CD8+GFP+ cells were sorted via Cytek Aurora CS. For POSH^fl/fl^ GzmB-Cre, cells were stimulated with 20 nM OVA peptide, treated with 1x murine recombinant IL-2, and harvested on day 3 post stimulation. Cells were stained with α-CD8 and CD8+GFP- and CD8+GFP+ cells were sorted via Cytek Aurora CS.

### RT PCR

1 million cells were lysed and RNA was harvested according to manufacturer instructions (PureLink RNA Mini Kit ThermoFisher 12183018A). RT PCR was performed using SuperScript IV VILO Master Mix (ThermoFisher 11756050) according to manufacturer instructions. Finally, a PCR on the cDNA to identify the presence or absence of exon seven was run using the following primers: FWD – GTG ACC CCA CCC CCT AGC REV - CCT GTG ACG GGC GCC ACA.

### Western blots

Cells were lysed using RIPA buffer containing leupeptin, aprotinin, PMSF, Na_3_VO_4_, and NaF for 30 minutes on ice. Cells were spun at 13,000 rpm for 30 minutes followed by the addition of 4x Laemmli Sample buffer (Bio-Rad 1610747) containing 2-mercaptoethanol (Sigma 7522). Lysates from 2 million cells per well were run on a 10% SDS-PAGE gel, transferred to a membrane, and blocked in 5% Bovine Serum Albumin (Fisher BP-1600-100) in TBST for 1 hour. Membranes were probed with primary antibody diluted in 5% BSA in TBST at 4C overnight. Membranes were washed then probed with secondary antibodies in 5% BSA in TBST at room temperature for 1 hour. Membranes were imaged on the Bio-Rad ChemiDoc MP Imaging System and images were analyzed using ImageJ.

### Flow cytometry

Tissues harvested were processed into single-cell suspensions by smashing the tissue between two pieces of mesh using a 3 mL syringe. Cells were washed in 1xPBS then resuspended in 1x ACK red blood cell lysis buffer for 3 minutes followed by an additional wash. Cells were subsequently blocked in Fc blocker for 15 minutes at room temperature and washed in FACS buffer. Cells were then stained extracellularly with a cocktail of antibodies prepared in FACS buffer and incubated in the dark on ice for 30–40 minutes. For thymic cells, the CCR7 antibody was added separately and incubated at 37C for 30–40 minutes. Cells were then washed twice and resuspended in FACS buffer for flow cytometry. Cell counts were obtained using 123count eBeads (Invitrogen 01-1234-42). For intracellular staining, the BD Pharmagen Transcription Factor set was utilized following manufactures instructions. All flow cytometry was performed on the Cytek Aurora.

For phospho-S6 analysis, cells were stained extracellularly as described above. Cells were then fixed in 2% PFA at room temperature for 20 minutes then permeabilized in 90% ice cold methanol for 20 minutes at –20C. Cells were then stained with phospho-S6 antibody diluted in 0.5% BSA in 1xPBS for 30 minutes in the dark at room temperature. Cells were washed twice followed by analysis by flow cytometry.

### T cell *in vitro* stimulations

Spleens from 6-12-week-old mice were harvested and processed into single-cell suspensions in complete RPMI 1640 media (1mM sodium pyruvate, 1x MEM non-essential amino acids solution, 100units/mL penicillin, 100units/mL streptomycin, 2mM L-glutamine, 0.02mM 2-mercaptoethanol, and 10% heat-inactivated FBS) at a concentration of 5 million/mL. Polyclonal cells were stimulated with 1 ug/mL α-CD3 and 1 ug/mL α-CD28 and incubated at 37C for 4 days. OT1 cells were stimulated with 20 nM OVA peptide and incubated at 37C for 4 days. At 24 hours, murine recombinant IL-2 (50 U/mL) was added to the cell culture. A portion of cells were taken each day for phenotyping analysis by flow cytometry as described above.

For cytokine production, cells were stimulated as described above. On day 2 post stimulation, cells were restimulated with 10 ug/mL PMA and 100 ug/mL ionomycin in the presence of Golgi-Plug (BD Biosciences) for 5 hours. Cells were then harvested and stained as described above.

### VSV-OVA infection

Splenocytes from 6–12-week-old OT1 POSHfl/fl, OT1 POSHfl/fl CD4-Cre, and OT1 POSHfl/fl GzmB-Cre (CD45.2) mice were harvested and processed into single cells suspensions in 1x PBS+1%FBS. 100,000 CD8 T cells were adoptively transferred via retro orbital injection into B6 (CD45.1) mice. The next day, recipient mice were infected with 2 million PFU of VSV-OVA via tail vein injection. At indicated time points, tail bleeds were performed to collect blood for phenotyping by flow cytometry as described above. At the endpoint of the experiment, blood, spleen, lymph nodes, and kidney were harvested and processed into single-cell suspensions. Cells were then stained on ice for 30–40 minutes, washed, and resuspended in FACS buffer. All flow cytometry was performed on the Cytek Aurora.

### EG7-OVA cell culture

EG7-OVA cells were cultured in complete RPMI 1640 media (1mM sodium pyruvate, 1x MEM non-essential amino acids solution, 100units/mL penicillin, 100units/mL streptomycin, 2mM L-glutamine, 0.02mM 2-mercaptoethanol, and 10% heat-inactivated FBS). Cells were maintained in an incubator at 37C and 5% CO_2_ and cell media was changed every 2 days.

### Killing assay

Splenic CD8 T cells were harvested from 6–12-week-old mice and stimulated as described above. On day 3 post stimulation, 6 million EG7-OVA target cells were harvested and stained with Tag-It Violet Proliferation and Cell Tracking Dye (Biolegend) according to manufacture instructions. 6 million CD8 T cells were harvested and co-cultured with stained EG7-OVA target cells at indicated effector:target ratios (E:T) for 5 hours. Cells were then washed, then stained with 7-AAD in the dark on ice for 30 minutes. Target cell death was analyzed by flow cytometry performed on the Cytek Aurora.

### Statistics

p values were calculated with paired two-tailed T test, one-way ANOVA with Tukey *post-hoc* test, or two-way ANOVA with Tukey multiple comparisons as denoted in figure legends. All analyses were performed with Prism 9 software (GraphPad).

### Antibodies and reagents

The 123count eBeads (01–1234–42), BSA (BP1600-100), PureLink RNA Mini Kit (12183018A), SuperScript IV VILO Master Mix (11756050), and Live/Dead™ Aqua (L34957) were purchased from ThermoFisher.

CD4 BV785 (RM4-5, 100552), CD4 PE Fire 640 (GK1.5, 100482), CD4 PerCP-Cy5.5 (RM4-5, 100540), CD8 BV421 (53-6.7, 100756), CD8 BV785 (53-6.7, 100750), TCRβ BV605 (H57-597, 109241), CD69 APC-Cy7 (H1.2F3, 104526), CD25 APC-Cy7 (PC61, 102025), CD62L PE-Cy7 (MEL-14, 104418), CD44 BV605 (IM7, 103047), CD62L PE-Cy7 (MEL-14, 104418), CD25 APC-Cy7 (PC61, 102025), CD69 AF700 (H1.2F3, 104539), CD127 BV421 (A7R34, 135027), CD45.2 BV711 (104, 109847), KLRG1 PE/Dazzle 594 (2F1, 138424), Ki67 BV650 (11F6, 151215), Streptavidin BV650 (405232), and Tag-It Violet Proliferation and Cell Tracking Dye (425101) were purchased from BioLegend.

CD8 PerCP-Cy5.5 (53-6.7, 551162), CCR7 APC (4B12, 17-1971-81), MHC1 PE (AF6-88.5, 553570), CD69 PE-Cy7 (H1.2F3, 552879), CD8 AF700 (53-6.7, 557959), CD8 PerCP-Cy5.5 (53-6.7, 551162), CD45.2 AF700 (104, 56–0454–82), and S6 (pS235/pS236) (560433) were purchased from BD Biosciences.

α-POSH (YY5, sc-100815) and α-p-JNK Thr183/Tyr185 (sc-6254) were purchased from Santa Cruz Biotechnology.

α-p-p65 (S536) (3033), α-p65 (6956), α-p-Akt S473 (4060), α-Akt (2920), α-JNK (9252), α-p-IκBα S32 (2859), and α-IκBα (4814) were purchased from Cell Signaling.

Bio Rad.

α-actin (12004163), α-mouse 700 (12004158), and α-rabbit 520 (12005869) were purchased from Bio-Rad.

OVA peptide (SIINFEKL) was purchased from Biosynth.

## Data Availability

The raw data supporting the conclusions of this article will be made available by the authors, without undue reservation.
