# Supplementary material for: The POSH scaffold protein is essential for signal coordination leading to CD8 T cell differentiation and survival
Source: Front Immunol. 2025 Jul 2;16:1630599. doi: 10.3389/fimmu.2025.1630599 (PMC12263621; doi:10.3389/fimmu.2025.1630599)
Supplement: Supplementary file 1 [file DataSheet1.pdf]

Supp Fig 1

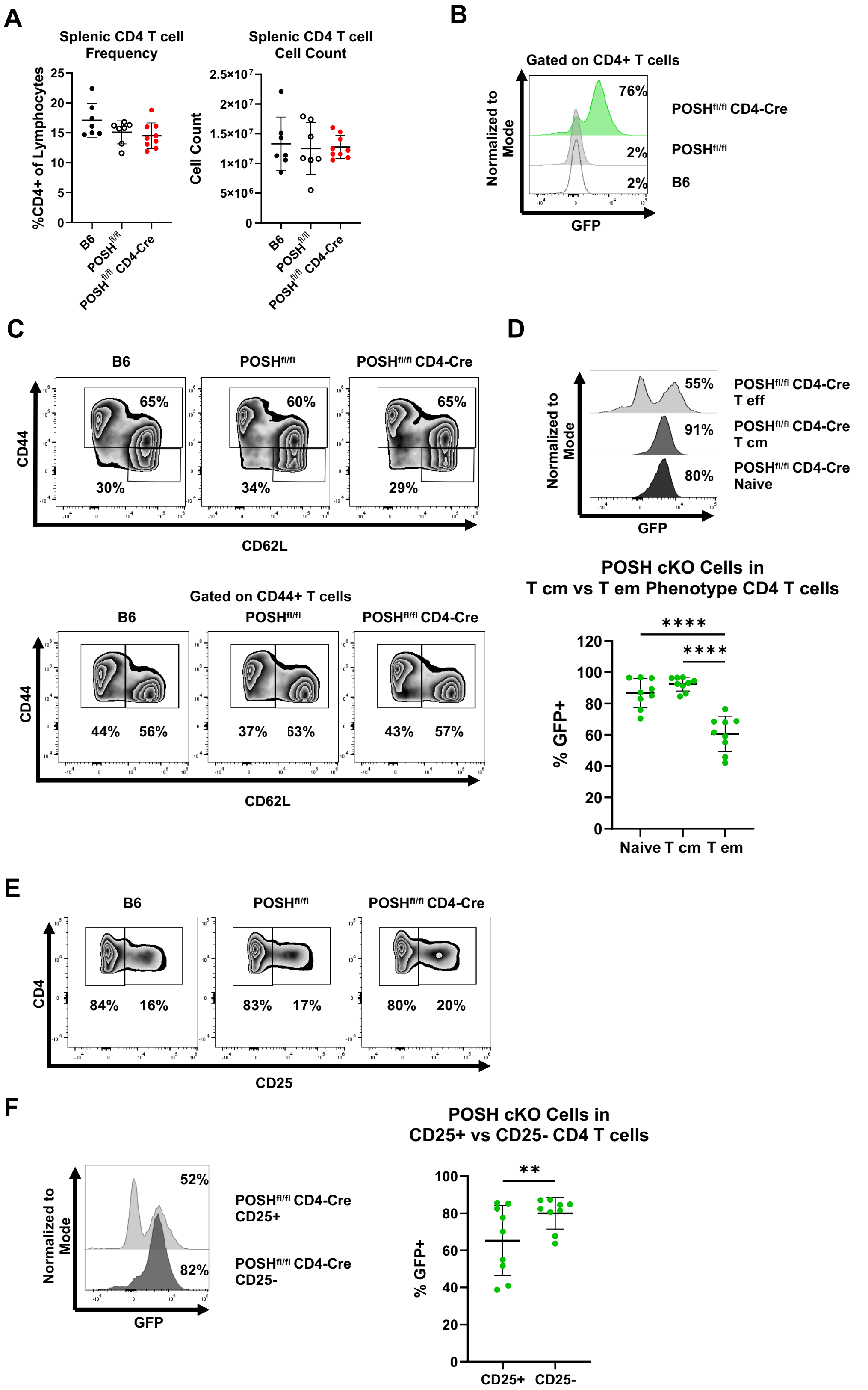

Supp Figure 1 POSH cKO CD4 T effector/effector memory cells are lost in POSH$^{fl/fl}$ CD4-Cre mice. A) Quantification of the frequency and number of CD4 T cells within B6, POSH$^{fl/fl}$, and POSH$^{fl/fl}$ CD4-Cre. B) Representative plot depicting the frequency of POSH cKO (GFP+) CD4+ T cells. C) Representative plots depicting the frequency of naïve (CD44-CD62L+) and CD44 high CD4+ T cells (top). Representative plots depicting the frequency of T cm (CD44+CD62L+) and T effector/effector memory (CD44+CD62L-) CD4 T cells (bottom). D) Representative plot (top) and quantification (bottom) of POSH cKO (GFP+) cells within the naïve, T cm, and T em CD4 T cell subsets in POSH$^{fl/fl}$ CD4-Cre mice. E) Representative plots depicting the frequency of CD25+ and CD25- CD4 T cells. F) Representative plot (left) and quantification (right) of POSH cKO (GFP+) cells within the CD25+ and CD25- CD4 T cell subsets in POSH$^{fl/fl}$ CD4-Cre mice. Data are shown as mean ± SD and are the combination of 3 independent experiments with n=7 B6, n=7 POSH$^{fl/fl}$, and n=9 POSH$^{fl/fl}$ CD4-Cre. Ordinary one-way ANOVA with Tukey's multiple comparison test, with a single pooled variance (A and D) or a paired two-tailed T test (F) was used to determine significance with *p<0.05, **p<0.001, ***p<0.0002, ****p<0.0001.

Supp Fig 2

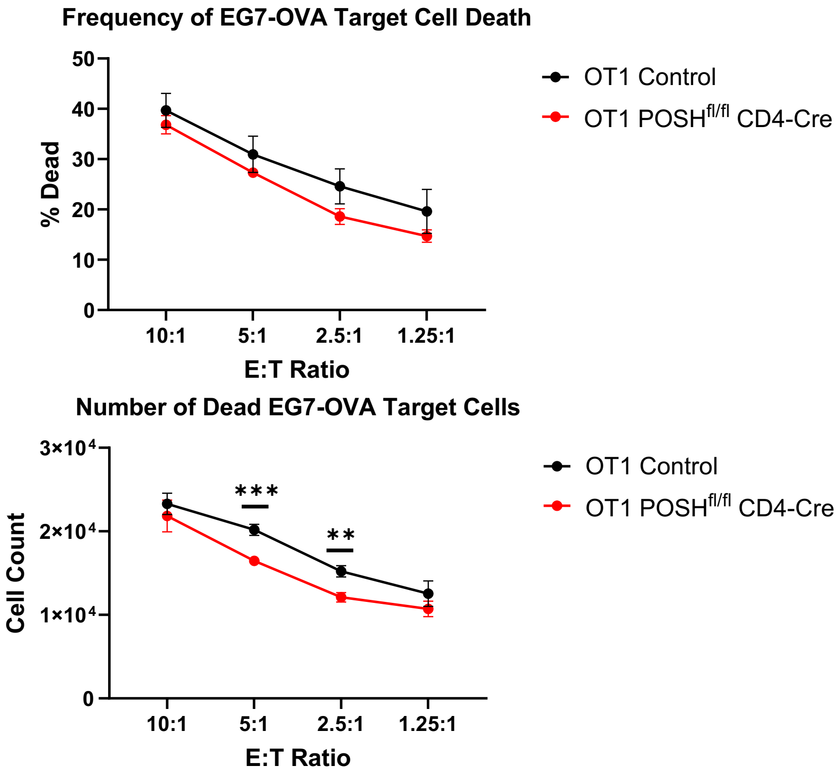

Supp Figure 2 POSH cKO CD8 T cells maintain the ability to kill target cells. OT1 Control and OT1 POSH$^{fl/fl}$ CD4-Cre cells were stimulated with OVA peptide for 3 days. Effector cells were then co-cultured with EG7-OVA target cells at indicated effector:target ratios (E:T) for 5 hours and target cell death was measured by 7-AAD staining A) Frequency (top) and number (bottom) of dead target cells in OT1 Control vs OT1 POSH$^{fl/fl}$ CD4-Cre co-cultures. Each E:T ratio was performed in triplicate and graphs depict the combination of 2 independent experiments. Multiple unpaired, two-tailed T test with Holm-Sidak's multiple comparisons test was used to determine significance with with *$p<0.05$, **$p<0.001$, ***$p<0.0002$, ****$p<0.0001$.

Supp Fig 3

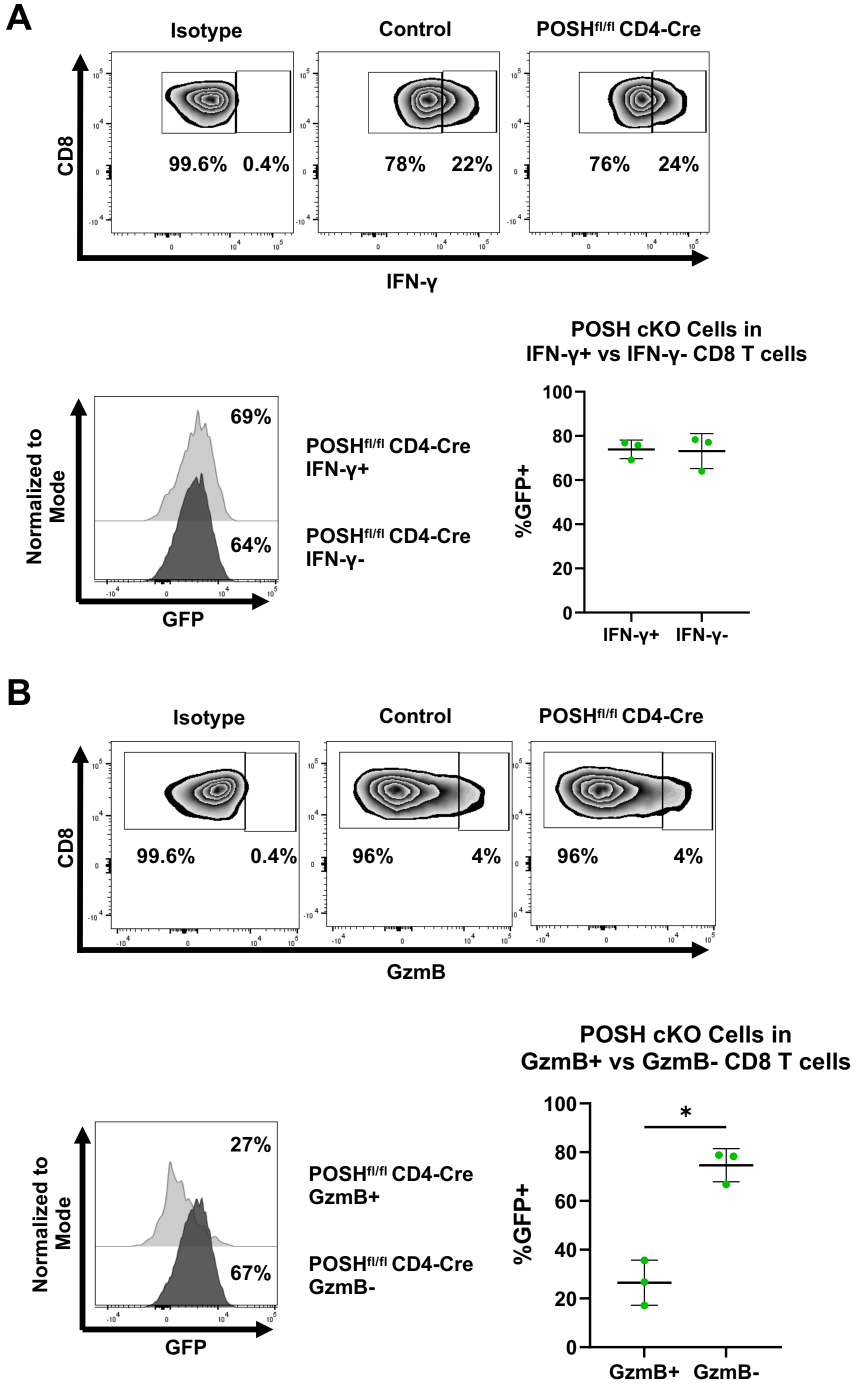

Supp Figure 3 POSH cKO CD8 T cells have decreased GzmB but not IFN-γ expression. Control and POSH$^{fl/fl}$ CD4-Cre CD8 T cells were stimulated with αCD3/αCD28 for 48 hours. Cells were there restimulated with PMA/Ionomycin for 5 hours and cytokine production was assessed. A) Representative plots depicting IFN-γ expression (top). Representative plot (bottom left) and quantification (bottom right) of the frequency of POSH cKO (GFP+) cells within the IFN-γ+ and IFN-γ- CD8 T cell subsets in POSH$^{fl/fl}$ CD4-Cre (n=3) cultures. B) Representative plots depicting GzmB expression (top). Representative plot (bottom left) and quantification (bottom right) of the frequency of POSH cKO (GFP+) cells within the GzmB+ and GzmB- CD8 T cell subsets in POSH$^{fl/fl}$ CD4-Cre (n=3) cultures. Data are shown as mean ± SD. Paired, two-tailed T test was used to determine significance with *p<0.05, **p<0.001, ***p<0.0002, ****p<0.0001.

Supp Fig 4

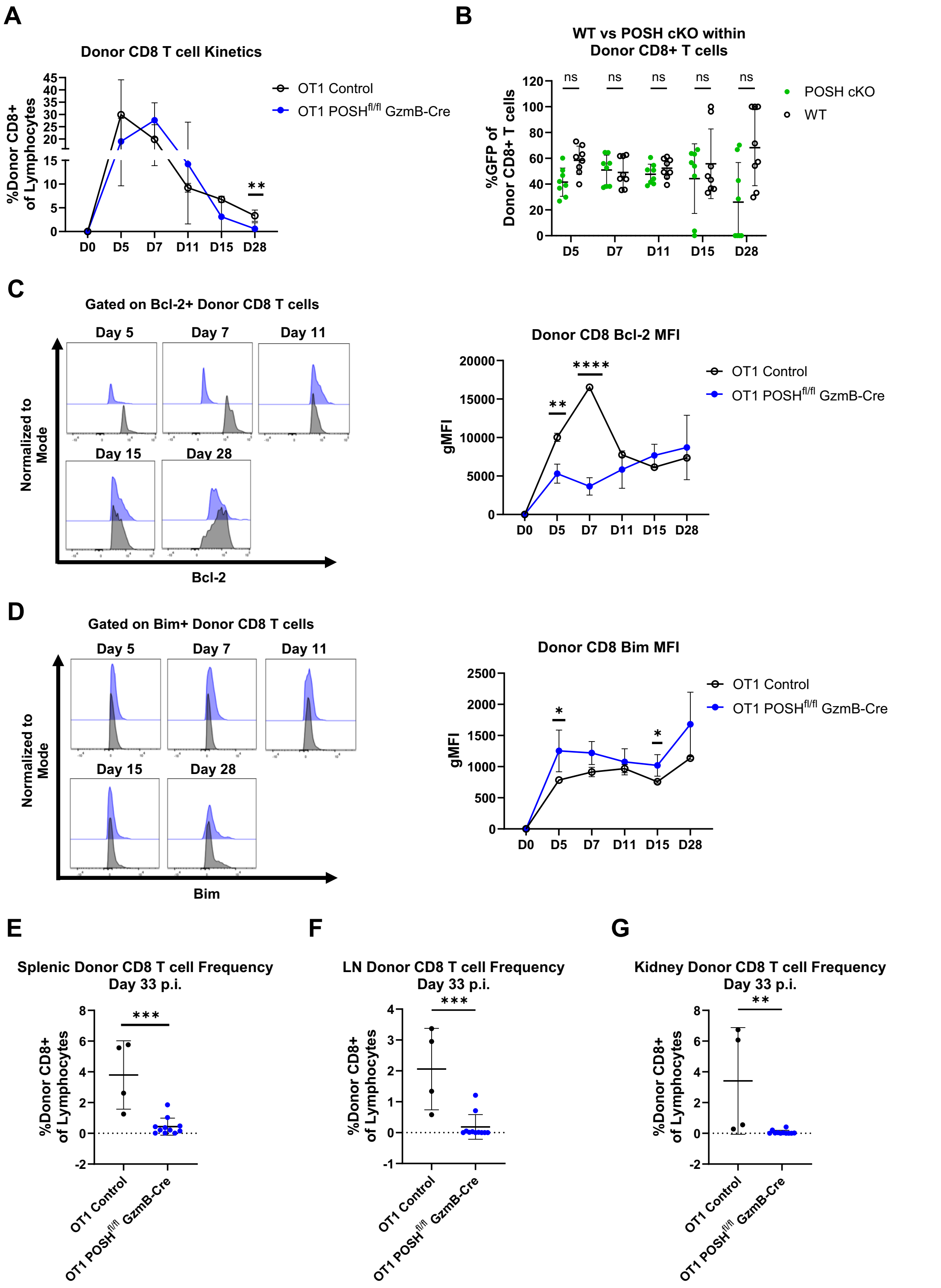

Supp Figure 4 POSH cKO donor cells are not equipped to survive into the memory phase post VSV-OVA infection. 100,000 OT1 Ly5.2 Control or OT1 Ly5.2 POSH$^{fl/fl}$ GzmB-Cre CD8 T cells were adoptively transferred into Ly5.1 B6 hosts. 24 hours later mice were injected i.v. with VSV-OVA. Tail bleeds were performed 5, 7, 11, 15, and 28-days post infection. 33 days post infection, mice were humanely euthanized and spleen, lymph node (LN) and kidney were harvested. A) Frequency of Ly5.2 CD8+ donor cells in the blood post VSV-OVA infection. n=4 OT1 Control and n=11 OT1 POSH$^{fl/fl}$ GzmB-Cre. B) Frequency of POSH cKO (GFP+) vs WT (GFP-) Ly5.2 CD8+ donor cells of mice adoptively transferred with OT1 POSH$^{fl/fl}$ GzmB-Cre cells (n=11). C-D) Representative plots (left) and quantification (right) of the gMFI of Bcl-2 (C) and Bim (D) within Ly5.2 CD8+ donor cells post VSV-OVA infection. n=2 OT1 Control and n=8 POSH$^{fl/fl}$ GzmB-Cre. E-G) Frequency of Ly5.2 CD8+ donor cells in the spleen (E), LN (F), and kidney (G) 33 days post VSV-OVA infection. n=4 OT1 Control, n=11 OT1 POSH$^{fl/fl}$ GzmB-Cre. Data are shown as mean ± SD and are the combination of 5 independent experiments. Significance was determined using A) Multiple unpaired T test with Holm-Sidak multiple comparisons test B) Multiple paired T test with Holm-Sidak's multiple comparisons C-D) Mixed-effects model with the Geisser-Greenhouse correction and Sidak's multiple comparisons test with individual variances computed for each comparison or E-G) Unpaired two-tailed T-test with *p<0.05, **p<0.001, ***p<0.0002, ****p<0.0001.

Supp Fig 5

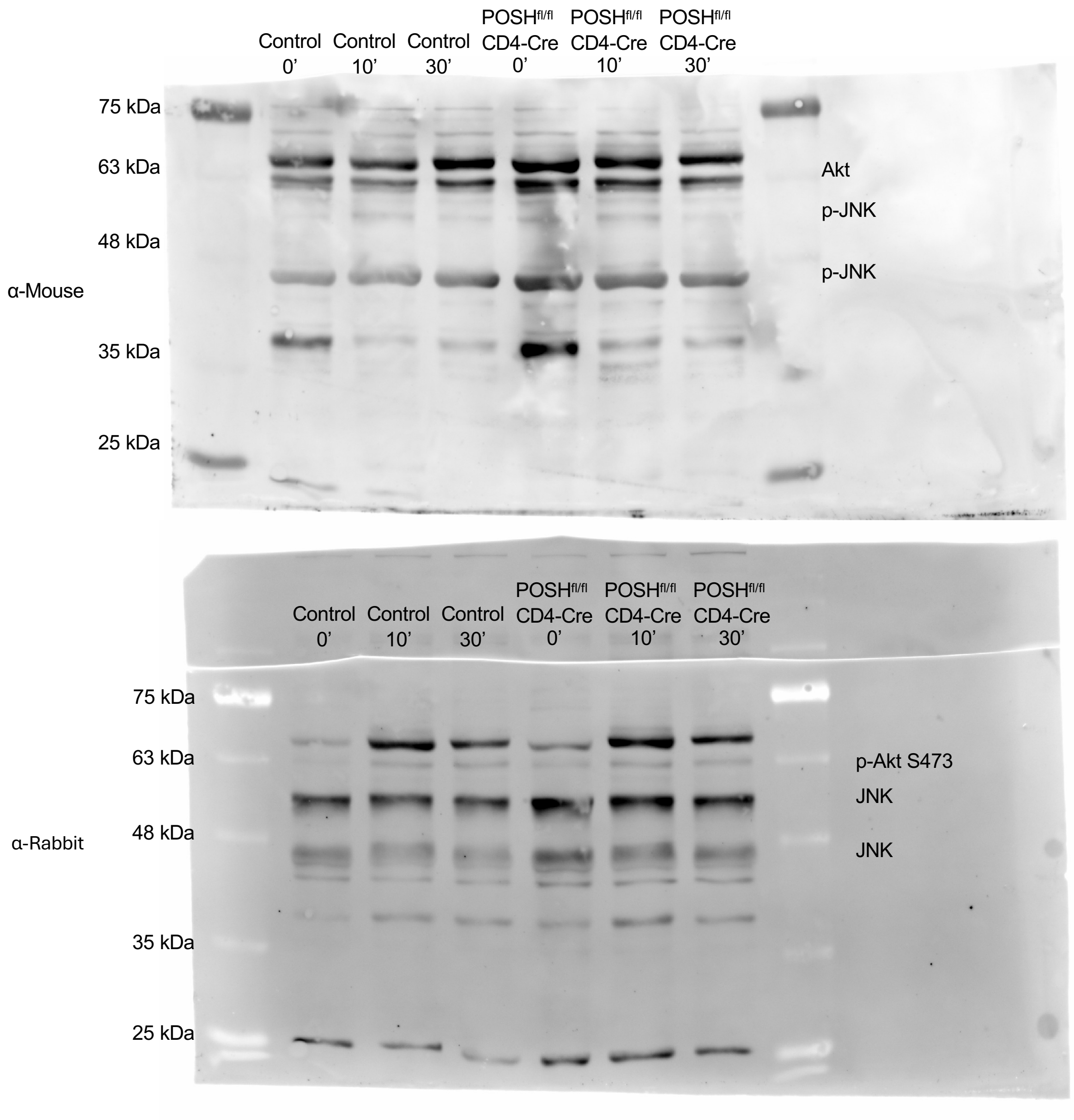

Supp Figure 5 Full Western Blots as shown in Figure 5 A and C

Supp Fig 6

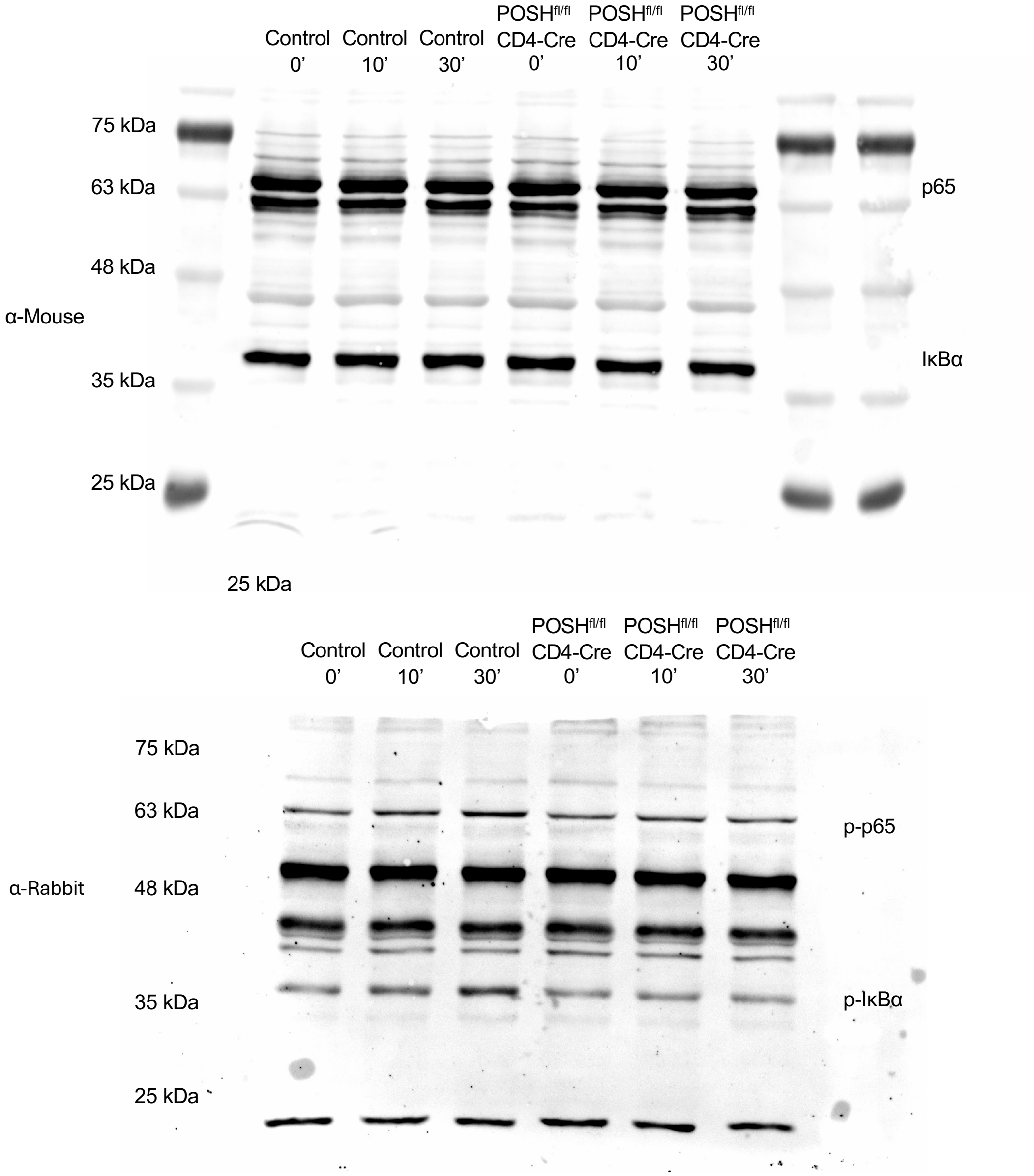

Supp Figure 6 Full Western Blots as shown in Figure 5B
